# An effective pan-serotype dengue vaccine and enhanced control strategies could help in reducing the severe dengue burden in Bangladesh–A perspective

**DOI:** 10.3389/fmicb.2024.1423044

**Published:** 2024-08-20

**Authors:** Mohammad Enamul Hoque Kayesh, Humayra Nazneen, Michinori Kohara, Kyoko Tsukiyama-Kohara

**Affiliations:** ^1^Department of Microbiology and Public Health, Faculty of Animal Science and Veterinary Medicine, Patuakhali Science and Technology University, Barishal, Bangladesh; ^2^Department of Haematology, Dhaka Medical College Hospital, Dhaka, Bangladesh; ^3^Department of Microbiology and Cell Biology, Tokyo Metropolitan Institute of Medical Science, Tokyo, Japan; ^4^Joint Faculty of Veterinary Medicine, Transboundary Animal Diseases Centre, Kagoshima University, Kagoshima, Japan

**Keywords:** dengue virus, dengue virus vaccine, severe dengue fever, epidemic, Bangladesh

## Abstract

Dengue is an important vector-borne disease occurring globally. Dengue virus (DENV) infection can result in a potentially life-threatening disease. To date, no DENV-specific antiviral treatment is available. Moreover, an equally effective pan-serotype dengue virus vaccine is not available. Recently, two DENV vaccines, Dengvaxia and Qdenga, were licensed for limited use. However, none of them have been approved in Bangladesh. DENV is transmitted by *Aedes* mosquitoes, and global warming caused by climate change favoring *Aedes* breeding plays an important role in increasing DENV infections in Bangladesh. Dengue is a serious public health concern in Bangladesh. In the year 2023, Bangladesh witnessed its largest dengue outbreak, with the highest number of dengue cases (*n* = 321,179) and dengue-related deaths (*n* = 1,705) in a single epidemic year. There is an increased risk of severe dengue in individuals with preexisting DENV-specific immunoglobulin G if the individuals become infected with different DENV serotypes. To date, vector control has remained the mainstay for controlling dengue; therefore, an immediate, strengthened, and effective vector control program is critical and should be regularly performed for controlling dengue outbreaks in Bangladesh. In addition, the use of DENV vaccine in curbing dengue epidemics in Bangladesh requires more consideration and judgment by the respective authority of Bangladesh. This review provides perspectives on the control and prevention of dengue outbreaks. We also discuss the challenges of DENV vaccine use to reduce dengue epidemics infection in Bangladesh.

## Introduction

Dengue, the most prevalent and rapidly spreading mosquito-borne viral disease in humans, is caused by the arbovirus, dengue virus (DENV) (Guzman et al., 2010). DENV belongs to the family *Flaviviridae* and the genus *Flavivirus* (Kuhn et al., 2002). A recent study suggested enzootic transmission of DENV in different animals, including pigs, non-human primates, marsupials, horses, bovids, rodents, dogs, other small animals, and birds (Gwee et al., 2021). DENV spreads through infected mosquitoes biting during a blood meal and exists in both sylvatic and urban ecosystems (Vasilakis et al., 2010). *Aedes aegypti* and *Aedes albopictus* mosquitoes are the primary vectors for DENV transmission, and the spread of DENV depends on the human-mosquito-human cycle through the bites of infectious female mosquitoes (Lambrechts et al., 2010). Vertical transmission of DENV in mosquitoes between generations has been observed, but its significance is yet to be established (Mulyatno et al., 2012; Grunnill and Boots, 2016).

DENV has four serotypes, DENV-1, DENV-2, DENV-3, and DENV-4, which are genetically and antigenically different (Simmons et al., 2012). DENV can cause a spectrum of illnesses in humans, ranging from self-resolving dengue fever to severe dengue hemorrhagic fever (DHF), and life-threatening dengue shock syndrome (DSS) in a small proportion of individuals (Harris et al., 2000). According to the new dengue case classification provided by the World Health Organization (WHO), severe plasma leakage leading to DSS and fluid accumulation with respiratory distress, severe bleeding, and severe organ involvement, including the liver, heart, and central nervous system, are features of severe dengue fever (Srikiatkhachorn, 2009; Ajlan et al., 2019; Htun et al., 2021). According to the recent estimation, 58–96 million symptomatic DENV infections occur annually, with over 10 million cases requiring hospitalization (Bhatt et al., 2013; Shepard et al., 2016).

Severe dengue is prevalent in areas where more than one DENV serotype co-circulates (Lorono-Pino et al., 1999; Guzman et al., 2010; Aguas et al., 2019). Although infection with one serotype can provide long-term protection against that particular serotype, only short-term (less than 6 months) protection is induced against infection with other serotypes (Rothman, 2011; Reich et al., 2013; Katzelnick et al., 2017; Salje et al., 2018). DHF is more common in patients with secondary DENV infections than in those with primary infection (Rothman, 2011). It has been reported that individuals are exposed to the greatest risk for developing severe dengue when they experience two sequential DENV infections with two different DENV types separated in time by more than 18 months (Montoya et al., 2013; Anderson et al., 2014). The global burden of DENV infections is increasing, with more than 400 million cases reported annually (Kariyawasam et al., 2023). This increasing trend is thought to continue, fueled by urbanization along with high population densities, human mobility, and climate change (Yang et al., 2021). An increasing trend of dengue burden has also been observed in Bangladesh, and recently, the dengue outbreak has increased many folds, which could be impacted by ineffective vector-control strategies, climate change, high population densities, and unplanned rapid urbanization and construction (Gubler, 2011; Lindsay et al., 2017; Kolimenakis et al., 2021; Gibb et al., 2023; Kayesh et al., 2023a). Bangladesh lies at a frequent/continuous level based on the dengue risk level set by the Centers for Disease Control and Prevention (United States) (Centers for Disease Control and Prevention, 2023).

The first dengue outbreak was reported in 1964 in Bangladesh, the then East Pakistan, and the term Dacca fever was coined (Russell et al., 1966). In 2000 Bangladesh experienced a large epidemic for the first time that resulted in 5,551 dengue cases and 93 deaths (Yunus et al., 2001). The epidemic in 2000 was likely due to introduction of a DENV strain probably from Thailand (Sharmin et al., 2015). Since then dengue has become endemic in Bangladesh, and dengue cases show a close relationship with the seasonal variation (Mutsuddy et al., 2019; Kayesh et al., 2023a). Among 40,476 dengue cases reported from 2000 to 2017, less than 1% (0.94%) were found in the pre-monsoon season, whereas most cases were found in the monsoon (50%) and post-monsoon (49%) season, and the peak period of dengue cases lies between July to October (Mutsuddy et al., 2019). Another study analyzed the 2,334 dengue cases that were diagnosed in Dhaka at a private diagnostic facility during 2010–2014, and it was reported that 90% of cases occurred between June and November (Morales et al., 2016).

Usually, dengue infection is extremely low in Bangladesh during the winter season (November to January), and no dengue cases were reported in Bangladesh in January to May during 2010 and 2012. However, since 2013, dengue cases have been reported during the pre-monsoon season (January to April), suggesting a change in dengue occurrence (Akbar et al., 2023). Notably, during 2023 outbreak 566 dengue cases were reported in the month of January only, suggesting the changes in the dengue outbreak timing, which could be attributed to multiple factors, including climatic change, e.g., unusual episodes of rainfall and outbreaks in neighboring countries and increased traveling (Akbar et al., 2023; Mahmud et al., 2024). A serological survey by Dhar-Chowdhury et al. in 2012 observed a higher dengue seroprevalence (93%) in Dhaka city among individuals tested during post-monsoon (Dhar-Chowdhury et al., 2017). The household utilities and water management practices and destruction of mosquito breeding sites and participation in mass gatherings were identified as important factors affecting dengue exposure (Dhar-Chowdhury et al., 2017).

**Figure 1:**
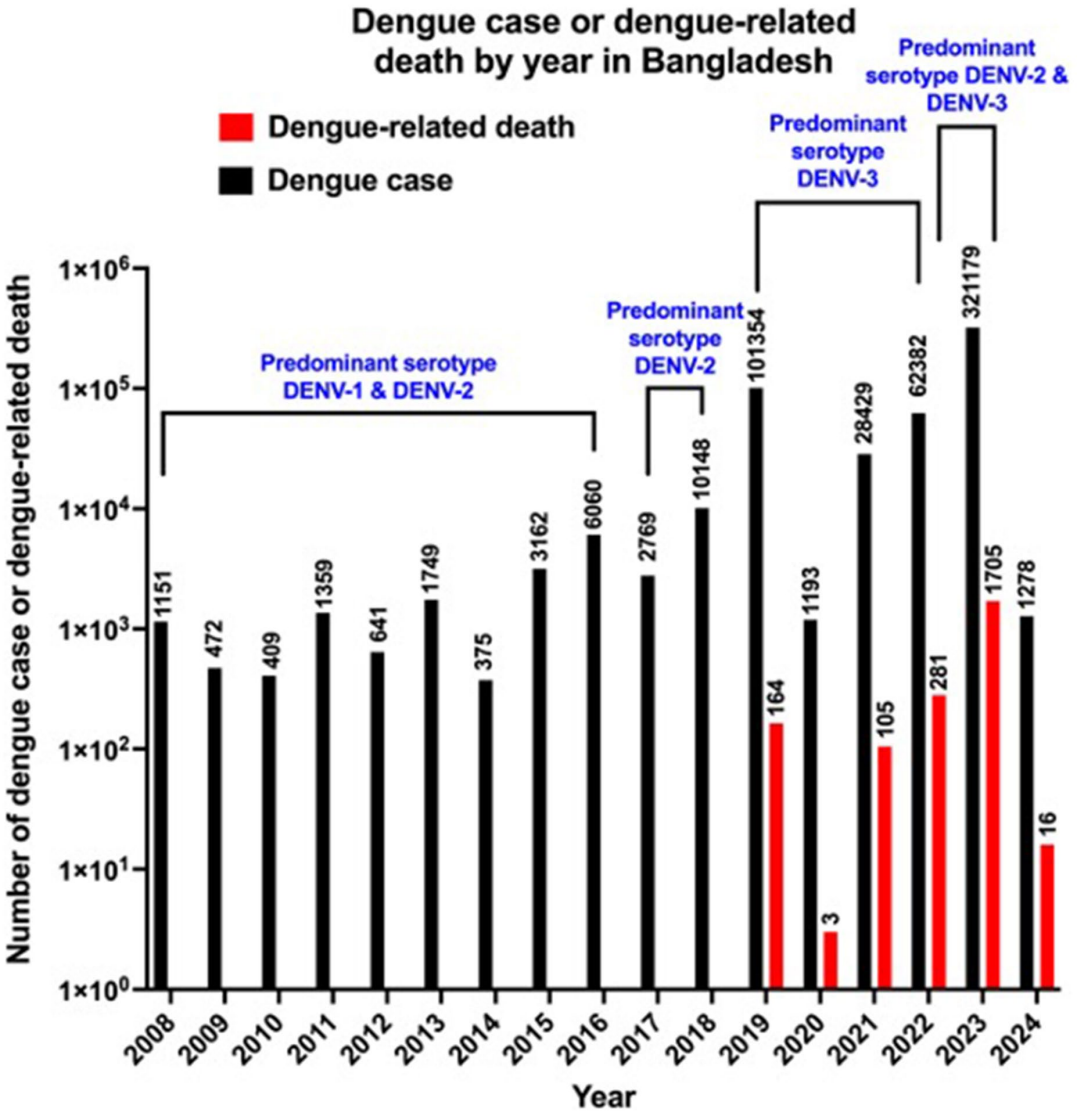
Number of reported dengue cases and dengue-related deaths per year in Bangladesh between 2008 and 2024. Data source: Directorate General of Health Services (DGHS) and the data for 2020 are limited due to COVID-19. The data for 2024 are partial (as of February 18, 2024).

In 2019, Bangladesh experienced one of the largest dengue epidemics in history with 101,354 dengue cases and 164 dengue-related deaths (Kayesh et al., 2023a). However, Bangladesh has witnessed another devastating dengue outbreak that has surpassed all the previous records of dengue epidemics, with record-breaking numbers of dengue cases (*n* = 321,179) and dengue-related deaths (*n* = 1,705) in a single epidemic year of 2023 (Figure 1) (Directorate General of Health Services of Bangladesh, 2023). Dengue outbreaks during 2000 to 2018 were mainly centered in Dhaka, however since 2019 dengue outbreaks spread to different districts of Bangladesh (Kayesh et al., 2023a; Sharif et al., 2024). Notably, during 2023 outbreak more than 50% of dengue cases were from outside of Dhaka (Sharif et al., 2024). There is variation in dengue prevalence based on sex, where a higher male incidence, compared to female has been observed in Bangladesh (Salje et al., 2019; Prattay et al., 2022). Similarly, a higher male incidence (60%) was reported among dengue-infected people, compared to female incidence (40%) during 2023 outbreak (Ashraf et al., 2024; Sharif et al., 2024). A higher incidence in male could be explained by part due to exposure of working-age males to outdoor environments when *Aedes aegypti* mosquitoes remain active (Yew et al., 2009; Prasith et al., 2013). A similar pattern of higher male incidence has previously been reported in four Asian countries, including Lao People’s Democratic Republic, the Philippines, Singapore and Sri Lanka (Anker and Arima, 2011). A difference in dengue incidence among different age groups has been observed during 2023 outbreak, where highest dengue cases (28.7%) were found among people aged 19–29 years and the second highest incidence (28.1%) was in 0–18 years group, and the lowest incidence (0.3%) was in over 80 years of age group (Sharif et al., 2024). Notably, despite fewer female cases in 2023 outbreak, the death incidence was significantly higher among females, accounting for 57% of total deaths, and the highest case fatality rate (12%) was in children aged between 0 and 10, suggesting the influence of sex and age differences on morbidity and mortality (Ashraf et al., 2024; Sharif et al., 2024).

Although dengue has no specific treatment, however, early diagnosis and proper clinical management can reduce the severity of dengue as well as dengue-related mortality (Tayal et al., 2023). Laboratory diagnosis of DENV infection can be done by direct detection of viral components (antigen detection) in the blood or indirect detection (antibody detection) by serological tests, depending on the day of illness (Kularatne and Dalugama, 2022). DENV infection causes alteration in vascular endothelial cell physiology and damage that lead to enhanced vascular permeability, inducing DSS (Basu and Chaturvedi, 2008). Early detection of the plasma leakage and appropriate fluid management remain key tool in management (Kularatne and Dalugama, 2022). Therefore, early clinical diagnosis and appropriate fluid treatment in plasma leakage may help in reducing DSS and dengue-related mortality (DENGUE Guidelines for diagnosis, treatment, prevention and control, 2009 WHO).

Although there are no panserotypes that are equally effective as dengue vaccines, there are two licensed dengue vaccines, Dengvaxia and Qdenga (Angelin et al., 2023; Thomas, 2023). However, no dengue vaccine has been approved for clinical use in Bangladesh. This new surge in dengue outbreaks indicates that the preventive measures taken to reduce dengue infections are insufficient, highlighting the necessity of further enhancement of preventive measures, as well as looking for new strategies, such as clinical trial of newly approved vaccine in Bangladeshi population for its suitability in Bangladesh. In this study, we provide a perspective on the enhancement of vector control programs and the things that need to be set/ensured before any approval of DENV vaccine use in Bangladesh.

## Strategies to reduce increased dengue burden in Bangladesh

To date, there is no specific treatment for dengue, and control of the vector is the key to dengue control and prevention. Dengue transmission shows high sensitivity to climatic conditions, especially temperature, rainfall and relative humidity (Naish et al., 2014; Kamal et al., 2023). It has been reported that the abundance and transmission potential of *Aedes aegypti* are influenced by both temperature and precipitation, indicating *Aedes* as a climate-sensitive vector (Johansson et al., 2009). Temperature and rainfall patterns are also critical in influencing the seasonal patterns of dengue transmission, where both wet conditions and extreme drought can increase the risk of dengue with different delays (Lowe et al., 2021).

It has been reported that the number of hospitalizations was increased in Dhaka, Bangladesh by hydro climatological variability, including both high and low river levels (Hashizume et al., 2012). Hossain et al. showed that factors such as temperature, humidity, and windspeed are critical in the transmission cycles of dengue disease, however, dengue cases reduced with higher levels of rainfall (Hossain et al., 2023b). In another study, Rahman et al. showed that a one extra rainy day in a month can increase dengue cases by 6% in the succeeding month (Rahman et al., 2020), suggesting the importance of developing a climate-based warning system in Bangladesh for dengue control. An extended duration of the peak dengue outbreak was observed in Bangladesh, which could have been due to climate change.

In a recent study, it has been shown that temperature is the dominant factor that shape dengue’s distribution and dynamics in Vietnam, as the warming enhances the transmission risk of dengue (Gibb et al., 2023). A recent study showed meteorological factors, including dew point, relative humidity, and rainfall influence the daily dengue cases in Bangladesh (Islam et al., 2023). The urban infrastructure factors, including sanitation, water supply, long-term urban growth as well as human mobility could enhance dengue emergence (Gibb et al., 2023). Urbanization is an important element linking with ecological, entomological, and epidemiological parameters that associate with the distribution and density of *Aedes* mosquitoes and thus disease transmission (Kolimenakis et al., 2021). Therefore, the control of rapid and unplanned urban expansion in Bangladesh is critical for curbing dengue epidemic. The climate conditions in Bangladesh are getting increasingly favorable for dengue transmission (Karim et al., 2012). Therefore, enhanced vector control programs are key tool in curbing the increased dengue burden in Bangladesh.

Although global collaborative efforts are required to strengthen dengue preparedness, prevention, and control, a strategy based on dengue outbreaks in the respective countries is essential to successfully reduce the outbreak and its spread. As dengue is a vector (*Aedes*)-borne disease, the control of *Aedes* mosquitoes is critical. Notably, other vector-borne diseases transmitted by mosquitoes, such as malaria and leishmaniasis, have been successfully controlled by adopting proper vector control programs (Wilson et al., 2020), highlighting the significance of implementing an efficient dengue vector control program that would be equally effective in reducing the dengue burden. Singapore has achieved great success in controlling dengue using an effective vector control program (Sim et al., 2020).

An effective vector-based DENV prevention is key for reducing increased dengue burden, and controlling vector should adopt multiple approaches, including reduction of breeding source, destruction of larvae by larvicide treatment, mass trapping, sleeping under nets, and killing adults using adulticides, etc. (Saied et al., 2015; Barrera, 2022; Kayesh et al., 2023a). There are different vector control strategies under the physical, chemical, and biological methods, targeting *Aedes aegypti* and *Aedes albopictus*, and the combination of different strategies is more effective than any single approach (Lima et al., 2015).

Tires, plastic drums, plastic buckets, and coconut shells are the most prevalent container types used for *Aedes* habitats in Bangladesh, and these habitat management (physical control), including proper use, disposal, and recycling of the containers are important for reducing the breeding site as well as for reducing the risk of DENV transmission (Paul et al., 2018; Rahman et al., 2021). In addition, to prevent birth of *Aedes* larvae in stagnant water every resident should regularly clean any accumulated water on the roof of the house, in the courtyard, or even in flower tubs. Chemical control in the form of fogging and spraying insecticides targeting adult mosquitoes and larvae are routinely practiced in Dhaka City, Bangladesh, however, these approaches remain to be effective in mosquito control (Eisen et al., 2009; Kayesh et al., 2023a). Insecticides belonging to different classes of chemical such as pyrethroids, carbamates, organophosphates, and organochlorines are used for chemical control of mosquitoes (Van Den Berg et al., 2012). A recent study reported the resistance of mosquitoes to pyrethroid insecticides, where all *Aedes aegypti* populations showed a high-level resistance to permethrin at the diagnostic dose, with mortality ranging from 0 to 14.8% and a substantial resistance to higher (2×) doses of permethrin, with mortality ranging from 5.1 to 44.4% (Al-Amin et al., 2020). Although complete susceptibility to bendiocarb has been observed in all populations (Al-Amin et al., 2020), this insecticide is not registered in Bangladesh, suggesting the need for alternate use of insecticides for effective mosquito control. Mosquitoes are also susceptible to malathion and deltamethrin, which have already been registered in Brazil and can be used as effective alternatives (Dhaka Tribune, 2019; Al-Amin et al., 2020).

Biological control approaches such as *Wolbachia*-mediated control (Khadka et al., 2020; Utarini et al., 2021), use of larvivorous fish (Seng et al., 2008; Paiva et al., 2014; Weeratunga et al., 2017), larvicidal toxins produced by *Bacillus thuringiensis* subspecies *israelensis* (Bti) and *Lysinibacillus sphaericus* (Huang et al., 2017), use of sterile insect technique (Oliva et al., 2012) could be investigated in Bangladesh for preventing the spread of DENV infection. However, Bangladesh needs to find out the most effective approach in the country context, and strengthen the vector control program by considering the extended duration of the peak dengue outbreak period, following proper national planning, as well as the dengue outbreak monitoring system. Additionally, it is equally important to educate people about the spread of DENV and how its spread could be restricted or reduced. The use of long sleeves can help avoid mosquito biting, which will ultimately help reduce the spread by reducing the number of biting sites.

Although there is much improvement in the approaches for promising efforts against DENV infection (Troost and Smit, 2020; Kaptein et al., 2021), there is still a lack of antivirals or immune-based (monoclonal antibodies) prophylactics or therapeutics against DENV, highlighting the necessity of enhancement of developing new strategies for achieving effective ways for controlling dengue. In 2023 outbreak, Bangladesh experienced record-breaking deaths due to dengue, as well as a number of dengue cases, warranting immediate effective and enhanced dengue vector control programs in the context of Bangladesh. Until an effective dengue vaccine against all four serotypes is available and approved, the enhancement of vector control strategies with regular monitoring of insecticide efficacy against *Aedes* mosquitoes is critical for improving the present dengue situation in Bangladesh. The local government engineering department is leading vector control activities, such as the elimination of mosquito breeding sites, the use of larvicides, and adult mosquito control using different insecticides, such as temephos and deltamethrin. The respective authorities involved in mosquito control programs should take immediate measures to determine the reasons for the rise in dengue vectors as well as to take measures for mosquito control.

Dengue is considered underreported in Bangladesh, as the dengue outbreak record is only maintained by passive surveillance, where the data are obtained from health facilities and community (Sharmin et al., 2018; Husain et al., 2019; Mamun et al., 2019; Hossain et al., 2023a), highlighting the necessity of introducing laboratory-based active surveillance system covering all hospitals/clinics and diagnostic labs in Dhaka city and outside the Dhaka (Hossain et al., 2023a). Serologic surveillance is important to know seroprevalence of the dengue in humans, which help in knowing how widespread transmission has occurred (Dhar-Chowdhury et al., 2017). Notably, DENV is expanding to new areas in Bangladesh, therefore the introduction of genomic surveillance in mosquito vector could help in tracking the prevalent DENV that might be involved in the subsequent dengue epidemics (Heralde et al., 2023; Maduranga et al., 2023). As DENV-infected people remain the source of spreading infection upon biting by mosquitoes in infected people; therefore, the restriction of mosquito biting to DENV-infected people is important, which could be maintained using mosquito curtain and mosquito-repellant cream on body surface both in infected and uninfected people. To limit the spread of DENV infection, restricted movement of DENV-infected people should be strictly maintained; if required, legislation should be enacted.

Another important aspect is that standard diagnostics and hospitals should be ensured peripherally at upazila (sub-district) and district level to restrict the movement of DENV-infected patients from the periphery to the center, which will reduce the spread of the disease.

Different diagnostic approaches can be used for dengue detection, including NS1 based antigen testing, IgM/IgG antibody testing, and polymerase chain reaction (Kabir et al., 2021). However, dengue is commonly diagnosed by serological assays in dengue diagnostic facilities in Bangladesh targeting DENV NS1 antigen (at early case), or dengue-specific IgM (at early/primary infection) or IgG (after 10–12 days of primary infection or at secondary infection) (Kabir et al., 2021; Lima et al., 2022). Although IgM or IgG based testing is useful to routine diagnosis of dengue infection, however the issues of low specificity and sensitivity cannot be excluded (Kabir et al., 2021). A PCR-based detection of the DENV infection provides more accurate results, which can be practiced in dengue diagnostic facilities of Bangladesh.

Community awareness is critical in the control of dengue vectors, which can be ensured by source reduction, elimination of container habitats favorable for oviposition sites, and permitting the development of aquatic stages by tightly fitting lids or covers on containers, or by killing larval and pupal stages using insecticides. Thus, community awareness campaigns could play an important role in educating people and help reduce breeding places for mosquitoes. Legislation can also be enacted to enhance community involvement in destroying or reducing mosquito breeding sites. Notably, both Dhaka South City Corporation and Dhaka North City Corporation operated mobile courts in houses and establishments so that water did not accumulate on the roof of the house, courtyard, or flower tub, and imposed fines to keep the households clean and free of mosquito larvae (Dhaka Tribune, 2023a,b). An effective vector control program and a strengthened monitoring system are unavoidable for curbing the dengue burden in Bangladesh.

## Recommended dengue vaccines for clinical use in individuals

A pan-serotype dengue vaccine with good efficacy has long been sought but is yet to be developed. As of February 18, 2024, there are two licensed, commercially available, live attenuated DENV vaccines sold under the brand names Dengvaxia and Qdenga. Dengvaxia, the first dengue vaccine, was developed by Sanofi Pasteur Co. and licensed in 2015 as a recombinant, live, attenuated, tetravalent dengue vaccine (CYD-TDV) (Hadinegoro et al., 2015). Yellow fever virus 17D was used as the backbone for Dengvaxia, and its prM/E RNA sequences were replaced with the corresponding prM/E RNA sequences of the DENV-1–4 serotypes (Guy et al., 2010). Dengvaxia was administered subcutaneously in three doses, and each dose was administered 6 months apart (at 0, 6, and 12 months) to obtain full protection. However, there are some limitations of Dengvaxia, such as age limitation (recommended for use in 9–45 years of age), recommendation for seropositive individuals (people who have been previously infected) only, increased risk of severe dengue development in seronegative participants, and serotype-dependent vaccine efficacy (Aguiar et al., 2016; Kayesh et al., 2021; Pintado Silva and Fernandez-Sesma, 2023), that have restrained its global use. Currently, Dengvaxia is available in only 20 countries (Pintado Silva and Fernandez-Sesma, 2023); however, it has not yet been approved in Bangladesh.

Takeda’s tetravalent dengue vaccine candidate (TAK-003) was based on live-attenuated DENV-2 (DEN2-PDK-53), which was originally designed and constructed by scientists at the Division of Vector-Borne Diseases of the US Centers for Disease Control and Prevention (CDC), providing a genetic backbone containing the three chimeric viruses containing the prM and E proteins of DENV-1, DENV-3, and DENV-4 (Huang et al., 2013; Osorio et al., 2016). TAK-003 was found to be effective against symptomatic dengue for over 3 years without any important safety risks in a 3-year long study of a population aged 4–16 years (*n* = 20,099) (Rivera et al., 2022). TAK-003 (Qdenga) is the second dengue vaccine to receive a license. It is a live, attenuated, and tetravalent dengue vaccine. The Qdenga vaccination course consisted of two injections administered 3 months apart. Phase III findings revealed that Qdenga appears encouraging compared to Dengvaxia (Thomas, 2023) (Table 1). Notably, in a phase III randomized controlled study, sequential or concomitant administration of the YF-17D vaccine and TAK-003 was found to be immunogenic and well tolerated, and non-inferiority of immune responses to YF-17D and TAK-003 was also observed for concomitant administration (Tricou et al., 2023).

**Table 1:** Comparison between two licensed dengue vaccines Dengvaxia and Qdenga.

Characteristic	Dengvaxia	Qdenga	References
Vaccine backbone	Yellow fever virus	DENV-2	Guy et al. (2010), Osorio et al. (2016)
Recommended age of vaccinee	16–45-year-old	4–65-year-old	Paz-Bailey et al. (2021), Angelin et al. (2023)
Number of doses	Three; each dose is administered 6 months apart	Two; each dose is administered 3 months apart	Aguiar et al. (2016), Pintado Silva and Fernandez-Sesma (2023), Thomas (2023), Tricou et al. (2024)
Route of administration	Subcutaneously	Subcutaneously	Paz-Bailey et al. (2021), Angelin et al. (2023), Tricou et al. (2024)
Recommended for seronegative individuals	No	Yes	Patel et al. (2023), Thomas (2023)
Recommended only in seropositive individuals	Yes	No	Wilder-Smith et al. (2019), Thomas (2023), Tricou et al. (2024)
Vaccine efficacy in seropositive individual	High	High	Thomas (2023), Tricou et al. (2024)
Antibody-dependent enhancement	Reported in seronegative individuals	–	Thomas (2023)
Protection against DENV serotypes	Provides only suboptimal protection against DENV-1 and DENV-2	Provides protection against DENV-1 and DENV-2, but not against DENV-3 in seronegative individuals. In case of DENV-4 in children the findings are not conclusive	Hou et al. (2020), Thomas (2023), Tricou et al. (2024)
Highest efficacy in phase 3 clinical study against DENV serotypes	DENV-4	DENV-2 (95.1%) [89.9–97.6]	Capeding et al. (2014), Biswal et al. (2020)
Prophylactic use	Not recommended	Possible	Lopez-Medina et al. (2022), Mukhtar et al. (2022), Biswal et al. (2023)
Vaccination efficacy varies by serotypes	Yes	Yes	Lopez-Medina et al. (2022), Rivera et al. (2022), Angelin et al. (2023)
Overall efficacy against symptomatic dengue	60%	73%	Hadinegoro et al. (2015), Lenharo (2023)
Safety profile	Low	High	Angelin et al. (2023), Biswal et al. (2023), Nascimento et al. (2023), Thomas (2023)

The Qdenga vaccine is recommended for use in children aged 4–60 years and in seronegative individuals, which could prevent all four serotypes. Qdenga has already received approval for use in Europe, the United Kingdom, Brazil, Argentina, Indonesia, and Thailand by the European Medicines Agency for individuals over 4 years of age and as per national recommendations (Angelin et al., 2023). Brazil is going to be the first country to include Qdenga in its public health system (Alves, 2024). However, the use of Qdenga has not yet been approved in Bangladesh. The WHO approved Qdenga on October 2, 2023, for emergency use to prevent dengue (Dhaka Tribune, 2023c). However, the dengue working group of the CDC Advisory Committee on Immunization Practices (ACIP) revealed that Qdenga did not protect against all serotypes equally, where better protection was reported in seronegative recipients against DENV-1 and-2 infection but comparatively less protection against DENV-3 (Thomas, 2023). Therefore, none of the existing dengue vaccines is sufficient to provide complete protection in DENV-seronegative individuals against all four serotypes (Odio et al., 2023). An overall comparison between Dengvaxia and Qdenga is summarized in Table 1.

Dengue-related deaths have sharply increased in Bangladesh, which is presumed to be linked to the prevalence and coinfection of multiple serotypes-induced antibody-dependent enhancement (ADE) effects among DENV-infected people (Katzelnick et al., 2017). Moreover, the association of host genetic factors and the influence of increased genetic diversity of DENV on severe dengue development cannot be excluded. Therefore, it is time to consider all necessary measures aimed at curbing the spread of the disease and tackling severe dengue fever and dengue-related deaths, including the increased infrastructure facilities for vaccine research as well as suitability of the future dengue vaccine uses in Bangladesh. Dengvaxia is safe and effective in reducing dengue-related hospitalizations and severe dengue in seropositive individuals. Accordingly, the ACIP has recommended the use of the Dengvaxia vaccine in the United States for children aged 9–16 years living in dengue endemic areas and with evidence of earlier dengue infection (Paz-Bailey et al., 2021). Bangladesh is also a dengue-endemic country, and many dengue epidemics may render numerous DENV-positive individuals.

Notably, it has been reported that the Bangladesh government is also considering dengue vaccine introduction, for which the advice of the National Immunization Technical Advisory Group of Bangladesh has been sought on the use of dengue vaccine in the country (Dhaka Tribune, 2023c). However, many issues such as how the vaccine strategy will work, who will cover the vaccine and vaccination cost, which age group of people will be vaccinated, availability of vaccines, the ADE-related risk of vaccination in improperly screened people due to poor diagnostic facilities, etc. are required to be finalized before any introduction of DENV vaccine in Bangladesh. Importantly, a globally licensed and effective pan-serotype dengue vaccine is still required.

## Dengue vaccines in different phases of clinical development

A pan-serotype-effective DENV vaccine that can inhibit DENV infection against all four serotypes in naïve individuals is yet to be developed. Moreover, ADE in the case of subprotective immunity poses a significant challenge to DENV vaccine development. However, many candidate vaccines are currently in different phases of development. TV003/TV005, a live attenuated tetravalent DENV vaccine candidate, was developed by the Laboratory of Infectious Diseases at the National Institutes of Health. Although TV003 and TV005 contain the same four monovalent components, they vary in dosing for serotype 2. TV005 contains a 10-fold higher dose of plaque-forming units to overcome the over attenuated serotype 2 component of TV003 (Whitehead, 2016). TV003/TV005 was found to be immunogenic and well tolerated, and administration of a single dose induced seroconversion to all four DENV serotypes in 74–92% (TV003) and 90% (TV005) of flavivirus-naive adults (Kirkpatrick et al., 2015; Whitehead et al., 2017). Notably, both the first and second doses (6 months apart) were well tolerated; however, no significant increase in antibody titers was observed after the booster dose (Kirkpatrick et al., 2015), suggesting that a single-dose regimen is likely to prevent DENV infection.

The vaccine candidates were licensed by several manufacturers for development, such as the Butantan Institute, Brazil, and a Phase II/III clinical trial was initiated (Durbin, 2020; Wilder-Smith, 2023). TV005 is the only single-dose tetravalent dengue vaccine, and a recent study evaluated the safety and immunogenicity of a single dose of TV005 across age groups in dengue-endemic Bangladesh (Walsh et al., 2023). To the best of our knowledge, this is the first study of a promising tetravalent dengue vaccine in dengue-endemic Bangladesh conducted by investigators from icddr,b, and the Larner College of Medicine at the University of Vermont (UVM). TV005 appears as good vaccine candidate and the efficacy, durability, and immune responses of this tetravalent dengue vaccine are ongoing worldwide (Walsh et al., 2023; Kallas et al., 2024). An ongoing phase 3, double-blind clinical trial in Brazil reported the overall 2 years vaccine efficacy findings in three different age groups (2–6 years, 7–17 years, and 18–59 years) (Kallas et al., 2024). It was observed that the highest efficacy was 90.0% (95% CI, 68.2–97.5) in the age group of 18–59 years, followed by 80.1% (95% CI, 66.0–88.4) in 2–6 years of age, and 77.8% (95% CI, 55.6–89.6) in 7–17 years of age group (Kallas et al., 2024). The vaccine efficacy differed between the serotypes, a higher efficacy (89.5, 95% CI, 78.7–95.0) was observed against DENV-1, compared to DENV-2 (69.6, 95% CI, 50.8–81.5), and DENV-3 and DENV-4 were undetected during the follow-up period (Kallas et al., 2024). Another recent study reported the findings of two randomized, controlled clinical trials of TV005, where this candidate vaccine was well tolerated and protected all vaccinated volunteers from viremia with DENV-2 or DENV-3 upon challenge with the rDEN2Δ30 or rDEN3Δ30 strain (Pierce et al., 2024), suggesting TV005 as a leading dengue vaccine candidate for further development.

In a Phase 1, double-blind, randomized, placebo-controlled study, a tetravalent live attenuated dengue vaccine manufactured in India was administered as a single subcutaneous injection in 60 healthy adults aged 18–45 years, revealing that the candidate vaccine was safe and well-tolerated (Gunale et al., 2023). The vaccine produced neutralizing antibodies and on day 57, the GMTs of neutralizing antibodies were between 66.76 (95% CI 36.63–121.69) to 293.84 (95% CI 192.25–449.11) for all four serotypes and was observed for 181 d. However, on day 181, the titers declined but were still much higher than the baseline (Gunale et al., 2023). Another Phase 1 study has been registered to evaluate the safety and immunogenicity of DENV-3 vaccination in a non-endemic population with the live attenuated recombinant DENV-3 monovalent dengue vaccine (rDEN3Δ30/31-7164) in seronegative, heterotypic (non-DENV-3), and more than one (polytypic) DENV serotype, which should provide information on immune responses upon primary, secondary, and tertiary exposure of DENV in naturally infected humans living in non-endemic areas (Trial registration: NCT05691530) (Odio et al., 2023).

The findings of a Phase 1 randomized clinical trial revealed that the V180 dengue vaccine candidate was safe and immunogenic in individuals who received the live attenuated tetravalent vaccine developed by the National Institute of Allergy and Infectious Diseases (Durbin et al., 2020). A phase I/II clinical trial of a cell culture-derived live-attenuated tetravalent dengue vaccine developed by Panacea Biotec Ltd. (PBL) was found to be safe and immunogenic in the Indian population (Mohanty et al., 2022). Although there was no statistically significant difference between the TDV and placebo groups in terms of AEs, the seroconversion rate in the TDV group was significantly higher (*p* < 0.001) than that in the placebo group (Mohanty et al., 2022). The seroconversion varied among vaccinated individuals against different serotypes, where among 92 individuals, 81.9% achieved seroconversion for DENV-1, 77.8% for DENV-2, 81.9% for DENV-3, and 79.2% for DENV-4 in TDV group (Mohanty et al., 2022).

## Dengue vaccines in preclinical development

Owing to the unsatisfactory nature of the efficacy and safety profiles of available DENV vaccines, a safe and effective dengue vaccine is required (Seesen et al., 2023). A recent study reported that the chimeric DENV-2/4EDII replicates efficiently *in vitro* and *in vivo*. A single inoculation of DENV-2/4EDII induced type-specific neutralizing antibodies against both DENV-2 and DENV-4 in male macaques (Young et al., 2023). In the rhesus model, a recombinant subunit vaccine candidate, V180, composed of DENV-truncated envelope (E) proteins (DEN-80E) for all four DENV serotypes, was found to induce strong neutralization titers that inhibited viremia even 8–12 months after the last vaccination (Govindarajan et al., 2015). In a randomized, placebo-controlled, Phase I study, nine V180 formulations were evaluated, where all formulations were found to be well tolerated in flavivirus-naïve adults (Manoff et al., 2019), and all V180 formulations with ISCOMATRI adjuvant induced robust immunogenicity compared to aluminum-adjuvanted and unadjuvanted formulations (Manoff et al., 2019).

To develop an effective DENV vaccine, scientists continue their efforts in many ways, and a recent study developed a DENV mRNA vaccine candidate encoding the membrane and envelope proteins from DENV-1 encapsulated in lipid nanoparticles (prM/E mRNA-LNP), which elicited robust antiviral immune responses with high levels of neutralizing antibody titers and antiviral CD4+ and CD8+ T cell responses in a mouse model (Wollner et al., 2021). In addition, after prM/E mRNA-LNP vaccination, AG129 mice showed protection against a lethal DENV challenge (Wollner et al., 2021), suggesting its efficacy for further development. In an *in vitro* study, another multitarget mRNA vaccine composed of DENV-1, DENV-2, DENV-3, DENV-4 envelope domain III (E-DIII), and non-structural protein 1 (NS1) coated with lipid nanoparticles induced a robust antiviral immune response and increased neutralizing antibody titers, inhibiting DENV infection in all serotypes without significant ADE (He et al., 2022).

Another study constructed three nucleotide-modified mRNA vaccine candidates for DENV-2, including prME-mRNA, E80-mRNA, and NS1-mRNA, providing complete protection against DENV-2 challenge in immunocompetent mice (Zhang et al., 2020). Vaccination with E80-mRNA alone or in combination with E80-mRNA and NS1-mRNA can induce high levels of neutralizing antibodies and antigen-specific T cell responses (Zhang et al., 2020). A recent study reported that a cyclic dinucleotide (CDN)-adjuvanted recombinant DENV NS1 vaccine induced serotype-specific and cross-reactive antibody and T cell responses in a mouse model (Espinosa et al., 2019). In addition, homotypic and heterotypic protection from DENV-2-induced morbidity and mortality has been observed (Espinosa et al., 2019). Another study reported that DENV-2 envelope domain III combined with TLR agonists could induce strong immunological signatures involving immune cell trafficking, interferons (IFNs), and pro-inflammatory and T-cell responses; however, it showed only partial protection against viral challenges (Bidet et al., 2019).

A DNA vaccine candidate consisting of the tandem envelope protein domain III (EDIII) of four dengue virus serotypes 1–4 and the DENV-2 NS1 protein-coding region has been reported to induce pan-serotype neutralizing antibodies and antigen-specific T cell responses in a mouse model (Sankaradoss et al., 2022). Moreover, it has been shown that the passive transfer of immune sera could protect AG129 mice against a virulent, non-mouse-adapted DENV-2 strain challenge (Sankaradoss et al., 2022), suggesting good efficacy of the vaccine and warrants for further development. Hou et al. showed that mice sequentially immunized with DNA vaccines encoding prM and E for four DENV serotypes (DENV-1to DENV-4) induced higher levels of neutralizing antibodies against all four DENV serotypes and significantly higher levels of IFNγ-or tumor necrosis factor (TNF)α-expressing CD4+ and CD8+ T cells than tetravalent vaccination (Hou et al., 2020). Moreover, similar neutralizing Ab responses were observed against all four DENV serotypes after the second to fourth immunizations, suggesting that ADE may not be a serious issue (Hou et al., 2020); however, further studies are required to confirm the effect of sequential immunization on ADE of that DNA candidate vaccine.

C57BL/6 mice vaccinated with plasmids encoding the DENV-2 non-structural proteins NS1, NS3, and NS5 provided complete protection against a DENV-2 strain naturally capable of inducing lethal encephalitis in immunocompetent mouse strains. Moreover, protection is correlated with the cytokine profiles expressed by the spleen and brain-infiltrating mononuclear cells (Alves et al., 2020). A DNA vaccine candidate expressing the prM and E proteins of DENV-3 was reported to induce strong antigen-specific T-cell responses and robust neutralizing antibodies (Feng et al., 2020). In addition, vaccinated mice showed long-term immunity against three other serotypes (Feng et al., 2020). Another DNA vaccine candidate expressing the prM-E protein of DENV-4 provided effective protection against a lethal DENV-4 challenge in immunocompetent BALB/c mice (Sheng et al., 2019).

Minatev et al. reported the development of a dengue virus vaccine containing DIII domains of the envelope protein of four DENV serotypes as vaccine antigens and modified vaccinia virus Ankara as the viral vector (Mintaev et al., 2023). All vaccinated mice showed a humoral response against all four dengue virus serotypes and virus-neutralizing activity against DENV (Mintaev et al., 2023). Our team developed a dengue vaccine candidate using a recombinant attenuated DIs strain of vaccinia virus containing NS proteins (NS2-5) of DENV2c (rDIs-NS25), which was found to induce effective cellular immunity in mice. We also evaluated the protective effects of rDIs-NS25 against DENV-1–4 serotypes in AG129 (interferon-alpha and gamma receptor double-knockout mice) and A129 (interferon-alpha knockout mice)-DENV infection systems [ISV2023, Kyoko-Tsukiyama-Kohara et al.].

## Challenges and prospects of clinical use of dengue virus vaccine in Bangladesh

Dengue is endemic to Bangladesh, and global warming and longer monsoon rains make it deadlier and one of the major public health concerns in Bangladesh (Walsh et al., 2023). The lack of an animal model that can comprehensively recapitulate human dengue infection remains a major challenge (Kayesh and Tsukiyama-Kohara, 2022), making it challenging to validate immune correlates of protection. Moreover, various risk factors, such as genetic background, sex, and other medical conditions including obesity, diabetes, and renal and cardiovascular diseases have been suggested to lead to the progression of severe dengue (Sangkaew et al., 2021). To date, no pan-serotype DENV vaccines have been approved. Moreover, the first licensed dengue vaccine, Dengvaxia, showed the inability to provide protection equally against all four serotypes; the least efficacy (42.3%) was reported in DENV-2 infection and the highest efficacy (77.4%) was against DENV-4 infection (Sabchareon et al., 2012). A 50.3% efficacy against DENV-1 and a 74.0% efficacy against DENV-3 infection was elicited by Dengvaxia (Sabchareon et al., 2012).

Managing a dengue outbreak in a tropical country, such as Bangladesh, is challenging, as favorable temperatures for mosquito breeding prevail almost year-round (Kayesh et al., 2023a). Moreover, Bangladesh is surrounded by two highly dengue endemic countries, including India and Myanmar that can cause increased viral transportation and transmission as well into Bangladesh (World Health Organization, 2023b). A recent study about dengue prevalence in Dhaka city reported the highest incidence of dengue in places with high ground temperature and lesser vegetation (Kamal et al., 2023). It has been reported that climatological circumstances such as higher temperatures, relative humidity, and precipitation can influence faster dengue transmission (Kamal et al., 2023). Another recent study also highlighted the increased local temperature and changes in rainfall seasonality contributing to increased dengue cases in Bangladesh (Hasan et al., 2024). In addition, air pollution may play an extra role in spreading dengue transmission (Lu et al., 2023) in Bangladesh. Moreover, the cross-reactivity of flaviviruses may lead to false diagnoses in clinical settings, resulting in delays in proper intervention and management. Therefore, accurate diagnosis with high specificity and sensitivity is essential to support the prompt and correct diagnosis of DENV infection (Kok et al., 2023). The genetic diversity of both DENV and its hosts poses another challenge in the use of the DENV vaccine; therefore, before introducing the vaccine, large efficacy trials demonstrating benefits across diverse populations in Bangladesh and clinical endpoints are warranted (Thomas, 2023). Because of ADE, each DENV serotype must be considered individually during vaccine development (Pintado Silva and Fernandez-Sesma, 2023). In Asia, DENV infection in children aged below 15 poses a higher risk of severe dengue development than in adults (Martina et al., 2009), and Qdenga use in children is supposed to reduce DENV infection as well as dengue-related severity. Therefore, the Bangladeshi government may consider Qdenga’s introduction as a new tool to mitigate dengue in countries like Bangladesh with a high disease burden (Wilder-Smith, 2024). However, according to one of the highest officials of the DGHS, the government of Bangladesh will follow a watch-and-see approach for any approval of Qdenga based on the efficacy of the vaccine in other markets.

Although severe DENV infections are not always associated with ADE and are preceded by infection with a heterologous serotype or increased viral load, many studies have demonstrated the role of ADE in DENV pathogenesis (Vaughn et al., 2000; Libraty et al., 2002; Wang et al., 2006; Simmons et al., 2007; Thomas et al., 2008; Teo et al., 2021, 2023b; Sawant et al., 2023). Similarly, the possibility of ADE exerted by the DENV vaccine could not be completely excluded. Therefore, before introducing a vaccine, it is critical to ensure the absence of ADE in vaccinated individuals. If there are any reports of vaccine-induced ADE effects on DENV infection, the vaccine must be stopped for use, and the DENV vaccine should only be recommended if there is no ADE in vaccinated individuals. However, a recent study in Mexico reported that individuals with at least two previous infections showed a comparatively lower risk of new infections than those in the seronegative group, suggesting the role of cross-immunity in protection (Amaya-Larios et al., 2020).

Although four DENV serotypes have been reported in Bangladesh, information on their evolution and genetic diversity remains limited (Suzuki et al., 2019). During 2000 and 2001 dengue outbreak in Bangladesh, DENV-3 was found as the most prevalent serotype, and that DENV-3 serotype was most closely related to DENV-3 emerged from neighboring Thailand and Myanmar but it was distinct from those from India and Sri Lanka (Podder et al., 2006). However, after that DENV-1 and DENV-2 were the predominant serotypes until 2016 (Figure 1) (Muraduzzaman et al., 2018; Ahsan et al., 2021). DENV-2 was the predominant serotype in 2017 and 2018 outbreak (Figure 1), which contained two distinct lineages of the DENV-2 Cosmopolitan genotype, and DENV-3 genotype I was found instead of DENV-3 genotype II, which was predominant during 2000 outbreak, indicating increased genetic diversity of DENV in Bangladesh (Suzuki et al., 2019; Rahim et al., 2023). DENV-3 genotype I was first detected in Bangladesh in 2017 (Rahim et al., 2023). Since 2019, DENV-3 has been the predominant serotype of dengue outbreak in Bangladesh (Figure 1) (Shirin et al., 2019; Rahim et al., 2021; World Health Organization, 2023a; Nafisa et al., 2024). In the 2023 outbreak, Bangladesh witnessed the highest number of dengue-related deaths (*n* = 1705) in a single epidemic year since the first recorded epidemic in 2000 (Directorate General of Health Services of Bangladesh, 2023). DENV-2, which is reported to be highly associated with severe form of dengue (Fried et al., 2010; Vicente et al., 2016), was identified as one of the predominant serotypes in the 2023 outbreak in Bangladesh (Figure 1) (World Health Organization, 2023a). Knowing which DENVs are circulating is important, however, there are limited information on the prevalent DENV serotypes in different outbreaks, suggesting a major gaps in surveillance system and early warning system (Haider et al., 2023); therefore, an adequate investigation of epidemiological research should be ascertained during any dengue outbreak, which could be helpful in predicting the dengue severity related to DENV serotypes and should help in early management in any future outbreak.

Dengue case fatality rates in Bangladesh from 2008 to 2024 (partial) are summarized in Table 2. There was a lack of dengue-related death from 2008 to 2018, which could be rendered by a lack of proper information/surveillance rather than the absence of dengue-related death. The case fatality rate (CFR) due to DENV infection has increased since dengue-related deaths were first recorded in 2019 (Table 2). The CFR for DENV infection in the 2019 outbreak was 0.16%, which is on the gradual increase, and more than three times higher (0.53%) in the 2023 outbreak and in the beginning of 2024, it appears 1.25% (as of 18 February, 2024) (Table 2), indicating a more severe recent dengue burden in Bangladesh. We cannot exclude the possibility of facing a more dangerous dengue situation in upcoming years if an effective vector control program is not implemented properly and in a timely manner. Moreover, the presence of DENV-4 as a predominant serotype may further worsen the future dengue epidemic in Bangladesh (Katzelnick et al., 2017; Kayesh et al., 2023a). Although Dengvaxia might be helpful in reducing the predicted death caused by DENV-4 infection, as Dengvaxia is highly effective against DENV-4, however, as Dengvaxia is recommended for seropositive individuals, avoiding the risk of vaccination in infection-naive individuals and ensuring the availability of a reliable DENV diagnostic tool in Bangladesh might appear to be a big challenge to overcome.

**Table 2:** Case fatality rate of DENV infection in Bangladesh.

Year	Case fatality rate (dengue-related death/dengue cases)
2008	0.0% (0/1,151)
2009	0.0% (0/472)
2010	0.0% (0/409)
2011	0.0% (0/1,359)
2012	0.0% (0/641)
2013	0.0% (0/1,749)
2014	0.0% (0/375)
2015	0.0% (0/3,162)
2016	0.0% (0/6,060)
2017	0.0% (0/2,769)
2018	0.0% (0/10,148)
2019	0.16% (164/1,01,354)
2020*	0.25% (3/1,193)
2021	0.37% (105/28,429)
2022	0.45% (281/62,382)
2023	0.53% (1705/3,21,179)
2024	1.25% (16/1,278)

Directorate General of Health Services (DGHS), Bangladesh (* data for 2020 are limited owing to COVID-19). The data for 2024 were collected on February 18, 2024.

## Future prospects

DENV has a unique and complex immunopathology that complicates dengue vaccine development. Moreover, the lack of suitable small animal models for immunopathogenesis studies and the absence of suitable markers of protective immunity are the major challenges in vaccine development that must be addressed (Ghosh and Dar, 2015; Kayesh et al., 2017). A large proportion of infected susceptible cells are infected with ADE, which can increase viremia and immunopathology (Wegman et al., 2023). An adequately reliable diagnostic tool with the desired sensitivity of 95% and specificity of 98% is essential for pre-vaccination screening to avoid any insufficient specificity and sensitivity in DENV detection, particularly in the detection of a previous monotypic dengue infection (Daag et al., 2021).

Sub-neutralizing or cross-reactive non-neutralizing antiviral antibodies remain a significant challenge in dengue vaccine development; therefore, it is important to develop an equally effective pan-serotype DENV vaccine lacking any ADE effect. TLR agonists can be investigated as dengue vaccine adjuvants to enhance the efficacy of dengue vaccines (Kayesh et al., 2023b). Wegman et al. demonstrated an association between DENV-reactive IgG and ADE in Fc gamma receptor-positive K562 cells. They further showed that IgA was not associated with ADE; rather, it effectively inhibited IgG-induced ADE activity (Wegman et al., 2021). Therefore, avoiding dengue vaccine-induced ADE is critical. Several studies (Goncalvez et al., 2007; Wang et al., 2017; Bournazos et al., 2020; Wegman et al., 2023; Teo et al., 2023a) have demonstrated the involvement of IgG in inducing ADE in DENV infection; therefore, the development of vaccines inducing DENV-specific cellular immunity to prevent DENV could provide a new platform for vaccine studies (ISV2023, Kyoko-Tsukiyama-Kohara et al.). As both the licensed DENV vaccines showed variable efficacy against different DENV serotypes and showed some limitations (Table 1), the ongoing efforts of different DENV vaccine development should be enhanced to have more suitable and effective vaccine candidate to battle DENV infection.

## Conclusion

Dengue is a significant threat to public health in Bangladesh. The dengue outbreak in 2023 crossed all previous records, causing the highest number of dengue cases (*n* = 321,179) and dengue-related deaths (*n* = 1,705), which is alarming. Insufficient and ineffective vector control programs may have enhanced the current dengue outbreaks. Therefore, an effective vector control program using the most effective insecticide needs to be established by the government and respective institutions as the top most priority for preventing DENV infection. The presence of multiple serotypes in Bangladesh remains a major threat to ADE-mediated dengue severity and the presence of DENV-2 as predominat serotype in 2023 outbreak may contribute increased dengue-related deaths. As the use of Qdenga in seronegative individuals and Dengvaxia in seropositive individuals seems promising, vaccines could be considered an important tool in reducing DENV infection-induced casualties in future dengue outbreaks in Bangladesh. However, before introducing vaccines in Bangladesh, nationwide mass trials with these vaccines among Bangladeshi people should be conducted. Moreover, the issues like the vaccine strategy, vaccine supply, vaccine cost, vaccinates age group, and ADE-related to vaccination should be taken into consideration for taking final judgment of vaccine use in controlling dengue epidemics. Finally, the government should follow the WHO guidelines and suggestions from experts in the country before taking the final step in introducing the dengue vaccine in Bangladesh.

## References

[ref1] Aguas R. Dorigatti I. Coudeville L. Luxemburger C. Ferguson N. M. (2019). Cross-serotype interactions and disease outcome prediction of dengue infections in Vietnam. Sci. Rep. 9:9395. doi: 10.1038/s41598-019-45816-6, PMID: 31253823 PMC6598999

[ref2] Aguiar M. Stollenwerk N. Halstead S. B. (2016). The risks behind Dengvaxia recommendation. Lancet Infect. Dis. 16, 882–883. doi: 10.1016/S1473-3099(16)30168-2, PMID: 27477967

[ref3] Ahsan A. Haider N. Kock R. Benfield C. (2021). Possible drivers of the 2019 dengue outbreak in Bangladesh: the need for a robust community-level surveillance system. J. Med. Entomol. 58, 37–39. doi: 10.1093/jme/tjaa150, PMID: 32725192

[ref4] Ajlan B. A. Alafif M. M. Alawi M. M. Akbar N. A. Aldigs E. K. Madani T. A. (2019). Assessment of the new World Health Organization's dengue classification for predicting severity of illness and level of healthcare required. PLoS Negl. Trop. Dis. 13:e0007144. doi: 10.1371/journal.pntd.0007144 31430283 PMC6716674

[ref5] Anderson K. B. Gibbons R. V. Cummings D. A. Nisalak A. Green S. Libraty D. H.. (2014). A shorter time interval between first and second dengue infections is associated with protection from clinical illness in a school-based cohort in Thailand. J. Infect. Dis. 209, 360–368. doi: 10.1093/infdis/jit436, PMID: 23964110 PMC3883164

[ref6] Akbar S. M. F. Khan S. Mahtab M. Mahtab M. A. Yahiro T. Arafat S. M.. (2023). Recent dengue infection in Bangladesh: A seasonal endemic progressing to year-long serious health concern. Euroasian J. Hepatogastroenterol. 13, 145–151. doi: 10.5005/jp-journals-10018-1408, PMID: 38222961 PMC10785144

[ref9] Al-Amin H. M. Johora F. T. Irish S. R. Hossainey M. R. H. Vizcaino L. Paul K. K.. (2020). Insecticide resistance status of *Aedes aegypti* in Bangladesh. Parasit. Vectors 13:622. doi: 10.1186/s13071-020-04503-6, PMID: 33317603 PMC7734861

[ref10] Alves L. (2024). Brazil to start widespread dengue vaccinations. Lancet 403:P133. doi: 10.1016/S0140-6736(24)00046-1, PMID: 38219747

[ref11] Alves R. Andreata-Santos R. De Freitas C. L. Pereira L. R. Fabris-Maeda D. L. N. Rodrigues-Jesus M. J.. (2020). Corrigendum: protective immunity to dengue virus induced by DNA vaccines encoding nonstructural proteins in a lethal challenge immunocompetent mouse model. Front. Med. Technol. 2:626114. doi: 10.3389/fmedt.2020.626114, PMID: 35051257 PMC8757852

[ref12] Amaya-Larios I. Y. Martinez-Vega R. A. Diaz-Quijano F. A. Sarti E. Puentes-Rosas E. Chihu L.. (2020). Risk of dengue virus infection according to serostatus in individuals from dengue endemic areas of Mexico. Sci. Rep. 10:19017. doi: 10.1038/s41598-020-75891-z, PMID: 33149151 PMC7642410

[ref13] Angelin M. Sjolin J. Kahn F. Ljunghill Hedberg A. Rosdahl A. Skorup P.. (2023). Qdenga(R) - A promising dengue fever vaccine; can it be recommended to non-immune travelers? Travel Med. Infect. Dis. 54:102598. doi: 10.1016/j.tmaid.2023.102598, PMID: 37271201

[ref14] Anker M. Arima Y. (2011). Male-female differences in the number of reported incident dengue fever cases in six Asian countries. Western Pac. Surveill. Response J. 2, e1–e23. doi: 10.5365/wpsar.2011.2.1.002 PMC3730962 23908884

[ref15] Ashraf S. Patwary M. M. Rodriguez-Morales A. J. (2024). Demographic disparities in incidence and mortality rates of current dengue outbreak in Bangladesh. New Microbes New Infect. 56:101207. doi: 10.1016/j.nmni.2023.101207, PMID: 38143942 PMC10746555

[ref16] Barrera R. (2022). New tools for Aedes control: mass trapping. Curr. Opin. Insect Sci. 52:100942. doi: 10.1016/j.cois.2022.100942 35667560 PMC9413017

[ref17] Basu A. Chaturvedi U. C. (2008). Vascular endothelium: the battlefield of dengue viruses. FEMS Immunol. Med. Microbiol. 53, 287–299. doi: 10.1111/j.1574-695X.2008.00420.x, PMID: 18522648 PMC7110366

[ref18] Bhatt S. Gething P. W. Brady O. J. Messina J. P. Farlow A. W. Moyes C. L.. (2013). The global distribution and burden of dengue. Nature 496, 504–507. doi: 10.1038/nature12060, PMID: 23563266 PMC3651993

[ref19] Bidet K. Ho V. Chu C. W. Naim A. N. H. Thazin K. Chan K. R.. (2019). Mimicking immune signatures of flavivirus infection with targeted adjuvants improves dengue subunit vaccine immunogenicity. NPJ Vaccines 4:27. doi: 10.1038/s41541-019-0119-3, PMID: 31285858 PMC6592935

[ref20] Biswal S. Borja-Tabora C. Martinez Vargas L. Velasquez H. Theresa Alera M. Sierra V.. (2020). Efficacy of a tetravalent dengue vaccine in healthy children aged 4-16 years: a randomised, placebo-controlled, phase 3 trial. Lancet 395, 1423–1433. doi: 10.1016/S0140-6736(20)30414-1 32197105

[ref21] Biswal S. Patel S. S. Rauscher M. (2023). Safety of dengue vaccine? Clin. Infect. Dis. 76, 771–772. doi: 10.1093/cid/ciac808, PMID: 36196620 PMC9938735

[ref22] Bournazos S. Gupta A. Ravetch J. V. (2020). The role of IgG fc receptors in antibody-dependent enhancement. Nat. Rev. Immunol. 20, 633–643. doi: 10.1038/s41577-020-00410-0, PMID: 32782358 PMC7418887

[ref23] Capeding M. R. Tran N. H. Hadinegoro S. R. Ismail H. I. Chotpitayasunondh T. Chua M. N.. (2014). Clinical efficacy and safety of a novel tetravalent dengue vaccine in healthy children in Asia: a phase 3, randomised, observer-masked, placebo-controlled trial. Lancet 384, 1358–1365. doi: 10.1016/S0140-6736(14)61060-6 25018116

[ref24] Centers for Disease Control and Prevention (2023). Dengue around the world. Available at: https://www.cdc.gov/dengue/areaswithrisk/around-the-world.html (Accessed August 20, 2023).

[ref25] Daag J. V. Ylade M. Adams C. Jadi R. Crisostomo M. V. Alpay R.. (2021). Evaluation of a new point-of-care test to determine prior dengue infection for potential use in pre-vaccination screening. Clin. Microbiol. Infect. 27, 904–908. doi: 10.1016/j.cmi.2020.08.026, PMID: 32866651

[ref26] Dhaka Tribune (2019). Majority of insecticides used by Dhaka city corporations ineffective against mosquitoes. Available at: https://www.dhakatribune.com/bangladesh/dhaka/182391/majority-of-insecticides-used-by-dhaka-city (Accessed September 15, 2023).

[ref27] Dhaka Tribune (2023a). Dengue danger: DSCC collects over 1L in fines in anti-mosquito drives. Available at: https://www.dhakatribune.com/bangladesh/dhaka/285959/dengue-danger-dscc-collects-over-1l-in-fines-in (Accessed October 19, 2023).

[ref28] Dhaka Tribune (2023b). Dengue: Four govt institutions fined 20L in anti-mosquito drives. Available at: https://www.dhakatribune.com/bangladesh/320109/dengue-four-govt-institutions-fined-20l-in (Accessed October 19, 2023).

[ref29] Dhaka Tribune (2023c). When will dengue vaccine Qdenga arrive in Bangladesh?. Available at: https://www.dhakatribune.com/bangladesh/health/328038/who-recommends-japan%E2%80%99s-%E2%80%98qdenga%E2%80%99-vaccine-for-dengue (Accessed October 19, 2023).

[ref30] Dhar-Chowdhury P. Paul K. K. Haque C. E. Hossain S. Lindsay L. R. Dibernardo A.. (2017). Dengue seroprevalence, seroconversion and risk factors in Dhaka, Bangladesh. PLoS Negl. Trop. Dis. 11:e0005475. doi: 10.1371/journal.pntd.0005475, PMID: 28333935 PMC5380355

[ref31] Directorate General of Health Services of Bangladesh (2023). Daily dengue status report. Available at: https://old.dghs.gov.bd/images/docs/vpr/20231231_dengue_all.pdf (Accessed February 18, 2024).

[ref32] Durbin A. P. (2020). Historical discourse on the development of the live attenuated tetravalent dengue vaccine candidate TV003/TV005. Curr. Opin. Virol. 43, 79–87. doi: 10.1016/j.coviro.2020.09.005, PMID: 33164790 PMC7685199

[ref33] Durbin A. P. Pierce K. K. Kirkpatrick B. D. Grier P. Sabundayo B. P. He H.. (2020). Immunogenicity and safety of a tetravalent recombinant subunit dengue vaccine in adults previously vaccinated with a live attenuated tetravalent dengue vaccine: results of a phase-I randomized clinical trial. Am. J. Trop. Med. Hyg. 103, 855–863. doi: 10.4269/ajtmh.20-0042 32394880 PMC7410446

[ref34] Eisen L. Beaty B. J. Morrison A. C. Scott T. W. (2009). ProactiveVector control strategies and improved monitoring and evaluation practices for dengue prevention. J. Med. Entomol. 46, 1245–1255. doi: 10.1603/033.046.0601, PMID: 19960667

[ref35] Espinosa D. A. Beatty P. R. Reiner G. L. Sivick K. E. Hix Glickman L. Dubensky T. W. Jr.. (2019). Cyclic dinucleotide-Adjuvanted dengue virus nonstructural protein 1 induces protective antibody and T cell responses. J. Immunol. 202, 1153–1162. doi: 10.4049/jimmunol.1801323, PMID: 30642979 PMC6363872

[ref36] Feng K. Zheng X. Wang R. Gao N. Fan D. Sheng Z.. (2020). Long-term protection elicited by a DNA vaccine candidate expressing the prM-E antigen of dengue virus serotype 3 in mice. Front. Cell. Infect. Microbiol. 10:87. doi: 10.3389/fcimb.2020.00087, PMID: 32257963 PMC7089926

[ref37] Fried J. R. Gibbons R. V. Kalayanarooj S. Thomas S. J. Srikiatkhachorn A. Yoon I. K.. (2010). Serotype-specific differences in the risk of dengue hemorrhagic fever: an analysis of data collected in Bangkok, Thailand from 1994 to 2006. PLoS Negl. Trop. Dis. 4:e617. doi: 10.1371/journal.pntd.0000617, PMID: 20209155 PMC2830471

[ref38] Ghosh A. Dar L. (2015). Dengue vaccines: challenges, development, current status and prospects. Indian J. Med. Microbiol. 33, 3–15. doi: 10.4103/0255-0857.148369, PMID: 25559995

[ref39] Gibb R. Colon-Gonzalez F. J. Lan P. T. Huong P. T. Nam V. S. Duoc V. T.. (2023). Interactions between climate change, urban infrastructure and mobility are driving dengue emergence in Vietnam. Nat. Commun. 14:8179. doi: 10.1038/s41467-023-43954-0, PMID: 38081831 PMC10713571

[ref40] Goncalvez A. P. Engle R. E. St Claire M. Purcell R. H. Lai C. J. (2007). Monoclonal antibody-mediated enhancement of dengue virus infection *in vitro* and *in vivo* and strategies for prevention. Proc. Natl. Acad. Sci. USA 104, 9422–9427. doi: 10.1073/pnas.0703498104 17517625 PMC1868655

[ref41] Govindarajan D. Meschino S. Guan L. Clements D. E. Ter Meulen J. H. Casimiro D. R.. (2015). Preclinical development of a dengue tetravalent recombinant subunit vaccine: immunogenicity and protective efficacy in nonhuman primates. Vaccine 33, 4105–4116. doi: 10.1016/j.vaccine.2015.06.067, PMID: 26144900

[ref42] Grunnill M. Boots M. (2016). How important is vertical transmission of dengue viruses by mosquitoes (Diptera: Culicidae)? J. Med. Entomol. 53, 1–19. doi: 10.1093/jme/tjv168, PMID: 26545718

[ref43] Gubler D. J. (2011). Dengue, urbanization and globalization: the unholy trinity of the 21(st) century. Trop. Med. Health 39, S3–S11. doi: 10.2149/tmh.2011-S05 PMC3317603 22500131

[ref44] Gunale B. Farinola N. Yeolekar L. Shrivastava S. Girgis H. Poonawalla C. S.. (2023). A phase 1, double-blind, randomized, placebo-controlled study to evaluate the safety and immunogenicity of a tetravalent live attenuated dengue vaccine in adults. Vaccine 41, 5614–5621. doi: 10.1016/j.vaccine.2023.07.045, PMID: 37532611

[ref45] Guy B. Guirakhoo F. Barban V. Higgs S. Monath T. P. Lang J. (2010). Preclinical and clinical development of YFV 17D-based chimeric vaccines against dengue, West Nile and Japanese encephalitis viruses. Vaccine 28, 632–649. doi: 10.1016/j.vaccine.2009.09.098, PMID: 19808029

[ref46] Guzman M. G. Halstead S. B. Artsob H. Buchy P. Farrar J. Gubler D. J.. (2010). Dengue: a continuing global threat. Nat. Rev. Microbiol. 8, S7–S16. doi: 10.1038/nrmicro2460, PMID: 21079655 PMC4333201

[ref47] Gwee S. X. W. St John A. L. Gray G. C. Pang J. (2021). Animals as potential reservoirs for dengue transmission: A systematic review. One Health 12:100216. doi: 10.1016/j.onehlt.2021.100216, PMID: 33598525 PMC7868715

[ref48] Hadinegoro S. R. Arredondo-Garcia J. L. Capeding M. R. Deseda C. Chotpitayasunondh T. Dietze R.. (2015). Efficacy and long-term safety of a dengue vaccine in regions of endemic disease. N. Engl. J. Med. 373, 1195–1206. doi: 10.1056/NEJMoa1506223 26214039

[ref49] Haider N. Asaduzzaman M. Hasan M. N. Rahman M. Sharif A. R. Ashrafi S. A. A.. (2023). Bangladesh's 2023 dengue outbreak - age/gender-related disparity in morbidity and mortality and geographic variability of epidemic burdens. Int. J. Infect. Dis. 136, 1–4. doi: 10.1016/j.ijid.2023.08.026 37660728

[ref50] Harris E. Videa E. Perez L. Sandoval E. Tellez Y. Perez M. L.. (2000). Clinical, epidemiologic, and virologic features of dengue in the 1998 epidemic in Nicaragua. Am. J. Trop. Med. Hyg. 63, 5–11. doi: 10.4269/ajtmh.2000.63.5, PMID: 11357995

[ref8] Hasan M. N. Khalil I. Chowdhury M. A. B. Rahman M. Asaduzzaman M. Billah M.. (2024). Two decades of endemic dengue in Bangladesh (2000-2022): trends, seasonality, and impact of temperature and rainfall patterns on transmission dynamics. J. Med. Entomol. 61, 345–353. doi: 10.1093/jme/tjae001, PMID: 38253990 PMC10936167

[ref51] Hashizume M. Dewan A. M. Sunahara T. Rahman M. Z. Yamamoto T. (2012). Hydroclimatological variability and dengue transmission in Dhaka, Bangladesh: a time-series study. BMC Infect. Dis. 12:98. doi: 10.1186/1471-2334-12-98, PMID: 22530873 PMC3528427

[ref52] He L. Sun W. Yang L. Liu W. Li J. (2022). A multiple-target mRNA-LNP vaccine induces protective immunity against experimental multi-serotype DENV in mice. Virol. Sin. 37, 746–757. doi: 10.1016/j.virs.2022.07.003, PMID: 35835315 PMC9583182

[ref53] Heralde F. M. III Obra G. M. Apelado M. P. B. (2023). “Genomic surveillance and intervention on dengue virus in an urban setting in the Philippines” in Dengue fever in a one health perspective-latest research and recent advances. ed. Sperança M. A. (Intechopen). doi: 10.5772/intechopen.109631

[ref54] Hossain S. Islam M. M. Hasan M. A. Chowdhury P. B. Easty I. A. Tusar M. K.. (2023b). Association of climate factors with dengue incidence in Bangladesh, Dhaka City: A count regression approach. Heliyon 9:e16053. doi: 10.1016/j.heliyon.2023.e16053, PMID: 37215791 PMC10192530

[ref55] Hossain M. S. Noman A. A. Mamun S. Mosabbir A. A. (2023a). Twenty-two years of dengue outbreaks in Bangladesh: epidemiology, clinical spectrum, serotypes, and future disease risks. Trop. Med. Health 51:37. doi: 10.1186/s41182-023-00528-6, PMID: 37434247 PMC10334535

[ref56] Hou J. Shrivastava S. Loo H. L. Wong L. H. Ooi E. E. Chen J. (2020). Sequential immunization induces strong and broad immunity against all four dengue virus serotypes. NPJ Vaccines 5:68. doi: 10.1038/s41541-020-00216-0, PMID: 32728482 PMC7382454

[ref57] Htun T. P. Xiong Z. Pang J. (2021). Clinical signs and symptoms associated with WHO severe dengue classification: a systematic review and meta-analysis. Emerg. Microbes Infect. 10, 1116–1128. doi: 10.1080/22221751.2021.1935327, PMID: 34036893 PMC8205005

[ref58] Huang Y. S. Higgs S. Vanlandingham D. L. (2017). Biological control strategies for mosquito vectors of arboviruses. Insects 8:21. doi: 10.3390/insects8010021 28208639 PMC5371949

[ref59] Huang C. Y. Kinney R. M. Livengood J. A. Bolling B. Arguello J. J. Luy B. E.. (2013). Genetic and phenotypic characterization of manufacturing seeds for a tetravalent dengue vaccine (DENVax). PLoS Negl. Trop. Dis. 7:e2243. doi: 10.1371/journal.pntd.0002243, PMID: 23738026 PMC3667780

[ref60] Husain M. Rahman M. Alamgir A. Uzzaman M. S. Flora M. S. (2019). Disease surveillance system of Bangladesh: combating public health emergencies. Online J. Public Health Inform. 11:e334. doi: 10.5210/ojphi.v11i1.9815

[ref7] Islam M. A. Hasan M. N. Tiwari A. Raju M. A. W. Jannat F. Sangkham S.. (2023). Correlation of dengue and meteorological factors in Bangladesh: A public health concern. Int. J. Environ. Res. Public Health 20:5152. doi: 10.3390/ijerph20065152 36982061 PMC10049245

[ref61] Johansson M. A. Dominici F. Glass G. E. (2009). Local and global effects of climate on dengue transmission in Puerto Rico. PLoS Negl. Trop. Dis. 3:e382. doi: 10.1371/journal.pntd.0000382, PMID: 19221592 PMC2637540

[ref62] Kabir M. A. Zilouchian H. Younas M. A. Asghar W. (2021). Dengue detection: advances in diagnostic tools from conventional technology to point of care. Biosensors (Basel) 11:206. doi: 10.3390/bios11070206 34201849 PMC8301808

[ref63] Kallas E. G. Cintra M. A. T. Moreira J. A. Patino E. G. Braga P. E. Tenorio J. C. V.. (2024). Live, attenuated, tetravalent Butantan-dengue vaccine in children and adults. N. Engl. J. Med. 390, 397–408. doi: 10.1056/NEJMoa2301790 38294972

[ref64] Kamal A. Al-Montakim M. N. Hasan M. A. Mitu M. M. P. Gazi M. Y. Uddin M. M.. (2023). Relationship between urban environmental components and dengue prevalence in Dhaka City-An approach of spatial analysis of satellite remote sensing, hydro-climatic, and census dengue data. Int. J. Environ. Res. Public Health 20:3858. doi: 10.3390/ijerph20053858 36900868 PMC10001735

[ref65] Kaptein S. J. F. Goethals O. Kiemel D. Marchand A. Kesteleyn B. Bonfanti J. F.. (2021). A pan-serotype dengue virus inhibitor targeting the NS3-NS4B interaction. Nature 598, 504–509. doi: 10.1038/s41586-021-03990-6 34671169

[ref66] Karim M. N. Munshi S. U. Anwar N. Alam M. S. (2012). Climatic factors influencing dengue cases in Dhaka city: a model for dengue prediction. Indian J. Med. Res. 136, 32–39, PMID: 22885261 PMC3461715

[ref67] Kariyawasam R. Lachman M. Mansuri S. Chakrabarti S. Boggild A. K. (2023). A dengue vaccine whirlwind update. Ther. Adv. Infect. Dis. 10:204993612311672. doi: 10.1177/20499361231167274 PMC10126642 37114191

[ref68] Katzelnick L. C. Gresh L. Halloran M. E. Mercado J. C. Kuan G. Gordon A.. (2017). Antibody-dependent enhancement of severe dengue disease in humans. Science 358, 929–932. doi: 10.1126/science.aan6836, PMID: 29097492 PMC5858873

[ref69] Kayesh M. E. H. Khalil I. Kohara M. Tsukiyama-Kohara K. (2023a). Increasing dengue burden and severe dengue risk in Bangladesh: An overview. Trop. Med. Infect. Dis. 8:8. doi: 10.3390/tropicalmed8010032 PMC9866424 36668939

[ref70] Kayesh M. E. H. Kitab B. Sanada T. Hayasaka D. Morita K. Kohara M.. (2017). Susceptibility and initial immune response of *Tupaia belangeri* cells to dengue virus infection. Infect. Genet. Evol. 51, 203–210. doi: 10.1016/j.meegid.2017.04.003, PMID: 28392469

[ref71] Kayesh M. E. H. Kohara M. Tsukiyama-Kohara K. (2021). Recent insights into the molecular mechanism of toll-like receptor response to dengue virus infection. Front. Microbiol. 12:744233. doi: 10.3389/fmicb.2021.744233, PMID: 34603272 PMC8483762

[ref72] Kayesh M. E. H. Kohara M. Tsukiyama-Kohara K. (2023b). TLR agonists as vaccine adjuvants in the prevention of viral infections: an overview. Front. Microbiol. 14:1249718. doi: 10.3389/fmicb.2023.1249718, PMID: 38179453 PMC10764465

[ref73] Kayesh M. E. H. Tsukiyama-Kohara K. (2022). Mammalian animal models for dengue virus infection: a recent overview. Arch. Virol. 167, 31–44. doi: 10.1007/s00705-021-05298-2, PMID: 34761286 PMC8579898

[ref74] Khadka S. Proshad R. Thapa A. Acharya K. P. Kormoker T. (2020). Wolbachia: a possible weapon for controlling dengue in Nepal. Trop. Med. Health 48:50. doi: 10.1186/s41182-020-00237-4, PMID: 32581639 PMC7310046

[ref75] Kirkpatrick B. D. Durbin A. P. Pierce K. K. Carmolli M. P. Tibery C. M. Grier P. L.. (2015). Robust and balanced immune responses to all 4 dengue virus serotypes following Administration of a Single Dose of a live attenuated tetravalent dengue vaccine to healthy, Flavivirus-naive adults. J. Infect. Dis. 212, 702–710. doi: 10.1093/infdis/jiv082, PMID: 25801652 PMC4612392

[ref76] Kok B. H. Lim H. T. Lim C. P. Lai N. S. Leow C. Y. Leow C. H. (2023). Dengue virus infection - a review of pathogenesis, vaccines, diagnosis and therapy. Virus Res. 324:199018. doi: 10.1016/j.virusres.2022.199018, PMID: 36493993 PMC10194131

[ref77] Kolimenakis A. Heinz S. Wilson M. L. Winkler V. Yakob L. Michaelakis A.. (2021). The role of urbanisation in the spread of Aedes mosquitoes and the diseases they transmit-A systematic review. PLoS Negl. Trop. Dis. 15:e0009631. doi: 10.1371/journal.pntd.0009631, PMID: 34499653 PMC8428665

[ref78] Kuhn R. J. Zhang W. Rossmann M. G. Pletnev S. V. Corver J. Lenches E.. (2002). Structure of dengue virus: implications for flavivirus organization, maturation, and fusion. Cell 108, 717–725. doi: 10.1016/S0092-8674(02)00660-8, PMID: 11893341 PMC4152842

[ref79] Kularatne S. A. Dalugama C. (2022). Dengue infection: global importance, immunopathology and management. Clin. Med. (Lond.) 22, 9–13. doi: 10.7861/clinmed.2021-0791, PMID: 35078789 PMC8813012

[ref80] Lambrechts L. Scott T. W. Gubler D. J. (2010). Consequences of the expanding global distribution of *Aedes albopictus* for dengue virus transmission. PLoS Negl. Trop. Dis. 4:e646. doi: 10.1371/journal.pntd.0000646, PMID: 20520794 PMC2876112

[ref81] Lenharo M. (2023). Dengue is spreading. Can new vaccines and antivirals halt its rise? Nature 623:470. doi: 10.1038/d41586-023-03453-0 37935865

[ref82] Libraty D. H. Endy T. P. Houng H. S. Green S. Kalayanarooj S. Suntayakorn S.. (2002). Differing influences of virus burden and immune activation on disease severity in secondary dengue-3 virus infections. J. Infect. Dis. 185, 1213–1221. doi: 10.1086/340365, PMID: 12001037

[ref83] Lima E. P. Goulart M. O. Rolim Neto M. L. (2015). Meta-analysis of studies on chemical, physical and biological agents in the control of *Aedes aegypti*. BMC Public Health 15:858. doi: 10.1186/s12889-015-2199-y, PMID: 26341708 PMC4559884

[ref84] Lima M. R. Q. Nunes P. C. G. Dos Santos F. B. (2022). Serological diagnosis of dengue. Methods Mol. Biol. 2409, 173–196. doi: 10.1007/978-1-0716-1879-0_12 34709642

[ref85] Lindsay S. W. Wilson A. Golding N. Scott T. W. Takken W. (2017). Improving the built environment in urban areas to control *Aedes aegypti*-borne diseases. Bull. World Health Organ. 95, 607–608. doi: 10.2471/BLT.16.189688, PMID: 28804174 PMC5537749

[ref86] Lopez-Medina E. Biswal S. Saez-Llorens X. Borja-Tabora C. Bravo L. Sirivichayakul C.. (2022). Efficacy of a dengue vaccine candidate (TAK-003) in healthy children and adolescents 2 years after vaccination. J. Infect. Dis. 225, 1521–1532. doi: 10.1093/infdis/jiaa761, PMID: 33319249 PMC9071282

[ref87] Lorono-Pino M. A. Cropp C. B. Farfan J. A. Vorndam A. V. Rodriguez-Angulo E. M. Rosado-Paredes E. P.. (1999). Common occurrence of concurrent infections by multiple dengue virus serotypes. Am. J. Trop. Med. Hyg. 61, 725–730. doi: 10.4269/ajtmh.1999.61.725 10586902

[ref88] Lowe R. Lee S. A. O'reilly K. M. Brady O. J. Bastos L. Carrasco-Escobar G.. (2021). Combined effects of hydrometeorological hazards and urbanisation on dengue risk in Brazil: a spatiotemporal modelling study. Lancet Planet Health 5, e209–e219. doi: 10.1016/S2542-5196(20)30292-8 33838736

[ref89] Lu H. C. Lin F. Y. Huang Y. H. Kao Y. T. Loh E. W. (2023). Role of air pollutants in dengue fever incidence: evidence from two southern cities in Taiwan. Pathog. Glob. Health 117, 596–604. doi: 10.1080/20477724.2022.2135711, PMID: 36262027 PMC10617642

[ref90] Maduranga S. Valencia B. M. Sigera C. Adikari T. Weeratunga P. Fernando D.. (2023). Genomic surveillance of recent dengue outbreaks in Colombo, Sri Lanka. Viruses 15:1408. doi: 10.3390/v15071408 37515097 PMC10384240

[ref91] Mahmud A. S. Bhattacharjee J. Baker R. E. Martinez P. P. (2024). Alarming trends in dengue incidence and mortality in Bangladesh. J. Infect. Dis. 229, 4–6. doi: 10.1093/infdis/jiad529, PMID: 38000901 PMC10786241

[ref92] Mamun M. A. Misti J. M. Griffiths M. D. Gozal D. (2019). The dengue epidemic in Bangladesh: risk factors and actionable items. Lancet 394, 2149–2150. doi: 10.1016/S0140-6736(19)32524-3, PMID: 31839186

[ref93] Manoff S. B. Sausser M. Falk Russell A. Martin J. Radley D. Hyatt D.. (2019). Immunogenicity and safety of an investigational tetravalent recombinant subunit vaccine for dengue: results of a phase I randomized clinical trial in flavivirus-naive adults. Hum. Vaccin. Immunother. 15, 2195–2204. doi: 10.1080/21645515.2018.1546523, PMID: 30427741 PMC6773383

[ref94] Martina B. E. Koraka P. Osterhaus A. D. (2009). Dengue virus pathogenesis: an integrated view. Clin. Microbiol. Rev. 22, 564–581. doi: 10.1128/CMR.00035-09, PMID: 19822889 PMC2772360

[ref95] Mintaev R. R. Glazkova D. V. Orlova O. V. Ignatyev G. M. Oksanich A. S. Shipulin G. A.. (2023). Development of MVA-d34 tetravalent dengue vaccine: design and immunogenicity. Vaccines (Basel) 11:831. doi: 10.3390/vaccines11040831 37112743 PMC10142911

[ref96] Mohanty L. Prabhu M. Kumar Mishra A. Purty A. J. Kanungo R. Ghosh G.. (2022). Safety and immunogenicity of a single dose, live-attenuated 'tetravalent dengue vaccine' in healthy Indian adults; a randomized, double-blind, placebo controlled phase I/II trial. Vaccine X 10:100142. doi: 10.1016/j.jvacx.2022.100142, PMID: 35252836 PMC8892502

[ref97] Montoya M. Gresh L. Mercado J. C. Williams K. L. Vargas M. J. Gutierrez G.. (2013). Symptomatic versus inapparent outcome in repeat dengue virus infections is influenced by the time interval between infections and study year. PLoS Negl. Trop. Dis. 7:e2357. doi: 10.1371/journal.pntd.0002357, PMID: 23951377 PMC3738476

[ref98] Morales I. Salje H. Saha S. Gurley E. S. (2016). Seasonal distribution and climatic correlates of dengue disease in Dhaka, Bangladesh. Am. J. Trop. Med. Hyg. 94, 1359–1361. doi: 10.4269/ajtmh.15-0846, PMID: 27114293 PMC4889757

[ref99] Mukhtar M. Wajeeha A. W. Zaidi N. Bibi N. (2022). Engineering modified mRNA-based vaccine against dengue virus using computational and reverse vaccinology approaches. Int. J. Mol. Sci. 23:13911. doi: 10.3390/ijms232213911, PMID: 36430387 PMC9698390

[ref100] Mulyatno K. C. Yamanaka A. Yotopranoto S. Konishi E. (2012). Vertical transmission of dengue virus in *Aedes aegypti* collected in Surabaya, Indonesia, during 2008-2011. Jpn. J. Infect. Dis. 65, 274–276. doi: 10.7883/yoken.65.274 22627316

[ref101] Muraduzzaman A. K. M. Alam A. N. Sultana S. Siddiqua M. Khan M. H. Akram A.. (2018). Circulating dengue virus serotypes in Bangladesh from 2013 to 2016. Virus 29, 303–307. doi: 10.1007/s13337-018-0469-x, PMID: 30159364 PMC6111961

[ref102] Mutsuddy P. Tahmina Jhora S. Shamsuzzaman A. K. M. Kaisar S. M. G. Khan M. N. A. (2019). Dengue situation in Bangladesh: An epidemiological shift in terms of morbidity and mortality. Can. J. Infect. Dis. Med. Microbiol. 2019, 1–12. doi: 10.1155/2019/3516284 PMC6431455 30962860

[ref103] Nafisa T. Akram A. Yeasmin M. Islam Resma T. Siddique M. A. B. Hosen N.. (2024). Predominant dengue virus serotype in Dhaka, Bangladesh: A research letter on samples from 2022 outbreak. Health Sci. Rep. 7:e1818. doi: 10.1002/hsr2.1818, PMID: 38250477 PMC10797646

[ref104] Naish S. Dale P. Mackenzie J. S. Mcbride J. Mengersen K. Tong S. (2014). Climate change and dengue: a critical and systematic review of quantitative modelling approaches. BMC Infect. Dis. 14:167. doi: 10.1186/1471-2334-14-167, PMID: 24669859 PMC3986908

[ref105] Nascimento E. J. M. Norwood B. Kpamegan E. Parker A. Fernandes J. Perez-Guzman E.. (2023). Antibodies produced in response to a live-attenuated dengue vaccine are functional in activating the complement system. J. Infect. Dis. 227, 1282–1292. doi: 10.1093/infdis/jiac476, PMID: 36461942 PMC10226660

[ref106] Odio C. D. Lowman K. E. Law M. Aogo R. A. Hunsberger S. Wood B. J.. (2023). Phase 1 trial to model primary, secondary, and tertiary dengue using a monovalent vaccine. BMC Infect. Dis. 23:345. doi: 10.1186/s12879-023-08299-5 37221466 PMC10204028

[ref107] Oliva C. F. Jacquet M. Gilles J. Lemperiere G. Maquart P. O. Quilici S.. (2012). The sterile insect technique for controlling populations of *Aedes albopictus* (Diptera: Culicidae) on Reunion Island: mating vigour of sterilized males. PLoS One 7:e49414. doi: 10.1371/journal.pone.0049414, PMID: 23185329 PMC3504010

[ref108] Osorio J. E. Wallace D. Stinchcomb D. T. (2016). A recombinant, chimeric tetravalent dengue vaccine candidate based on a dengue virus serotype 2 backbone. Expert Rev. Vaccines 15, 497–508. doi: 10.1586/14760584.2016.1128328, PMID: 26635182

[ref109] Paiva C. N. Lima J. W. Camelo S. S. Lima Cde F. Cavalcanti L. P. (2014). Survival of larvivorous fish used for biological control of *Aedes aegypti* (Diptera: Culicidae) combined with different larvicides. Trop. Med. Int. Health 19, 1082–1086. doi: 10.1111/tmi.12341, PMID: 24890120

[ref110] Patel S. S. Winkle P. Faccin A. Nordio F. Lefevre I. Tsoukas C. G. (2023). An open-label, phase 3 trial of TAK-003, a live attenuated dengue tetravalent vaccine, in healthy US adults: immunogenicity and safety when administered during the second half of a 24-month shelf-life. Hum. Vaccin. Immunother. 19:2254964. doi: 10.1080/21645515.2023.2254964, PMID: 37846724 PMC10583633

[ref111] Paul K. K. Dhar-Chowdhury P. Haque C. E. Al-Amin H. M. Goswami D. R. Kafi M. A. H.. (2018). Risk factors for the presence of dengue vector mosquitoes, and determinants of their prevalence and larval site selection in Dhaka, Bangladesh. PLoS One 13:e0199457. doi: 10.1371/journal.pone.0199457, PMID: 29928055 PMC6013170

[ref112] Paz-Bailey G. Adams L. Wong J. M. Poehling K. A. Chen W. H. McNally V.. (2021). Dengue vaccine: recommendations of the advisory committee on immunization practices, United States, 2021. DHHS Publ. 70, 1–16. doi: 10.15585/mmwr.rr7006a1, PMID: 34978547 PMC8694708

[ref113] Pierce K. K. Durbin A. P. Walsh M. R. Carmolli M. Sabundayo B. P. Dickson D. M.. (2024). TV005 dengue vaccine protects against dengue serotypes 2 and 3 in two controlled human infection studies. J. Clin. Invest. 134:e173328. doi: 10.1172/JCI173328, PMID: 37971871 PMC10836801

[ref114] Pintado Silva J. Fernandez-Sesma A. (2023). Challenges on the development of a dengue vaccine: a comprehensive review of the state of the art. J. Gen. Virol. 104:001831. doi: 10.1099/jgv.0.001831, PMID: 36857199 PMC10228381

[ref115] Podder G. Breiman R. F. Azim T. Thu H. M. Velathanthiri N. Mai Le Q.. (2006). Origin of dengue type 3 viruses associated with the dengue outbreak in Dhaka, Bangladesh, in 2000 and 2001. Am. J. Trop. Med. Hyg. 74, 263–265. doi: 10.4269/ajtmh.2006.74.263 16474082

[ref116] Prasith N. Keosavanh O. Phengxay M. Stone S. Lewis H. C. Tsuyuoka R.. (2013). Assessment of gender distribution in dengue surveillance data, the Lao People's Democratic Republic. Western Pac. Surveill. Response J. 4, 17–24. doi: 10.5365/WPSAR.2012.3.4.020, PMID: 24015367 PMC3762968

[ref117] Prattay K. M. R. Sarkar M. R. Shafiullah A. Z. M. Islam M. S. Raihan S. Z. Sharmin N. (2022). A retrospective study on the socio-demographic factors and clinical parameters of dengue disease and their effects on the clinical course and recovery of the patients in a tertiary care hospital of Bangladesh. PLoS Negl. Trop. Dis. 16:e0010297. doi: 10.1371/journal.pntd.0010297 35377886 PMC8979461

[ref118] Rahim R. Hasan A. Hasan N. Nakayama E. E. Shioda N. Rahman M. (2021). Diversity of dengue virus serotypes in Dhaka City: from 2017 to 2021. Bangladesh J. Med. Microbiol. 15, 23–29. doi: 10.3329/bjmm.v15i2.57817

[ref119] Rahim R. Hasan A. Phadungsombat J. Hasan N. Ara N. Biswas S. M.. (2023). Genetic analysis of dengue virus in severe and non-severe cases in Dhaka, Bangladesh, in 2018-2022. Viruses 15:1144. doi: 10.3390/v15051144 37243230 PMC10222234

[ref120] Rahman M. S. Faruk M. O. Tanjila S. Sabbir N. M. Haider N. Chowdhury S. (2021). Entomological survey for identification of Aedes larval breeding sites and their distribution in Chattogram, Bangladesh. Beni-Suef University J. Basic Appl. Sci. 10:32. doi: 10.1186/s43088-021-00122-x

[ref121] Rahman K. M. Sharker Y. Rumi R. A. Khan M. I. Shomik M. S. Rahman M. W.. (2020). An association between rainy days with clinical dengue fever in dhaka, Bangladesh: findings from a hospital based study. Int. J. Environ. Res. Public Health 17:9506. doi: 10.3390/ijerph17249506 33353025 PMC7765799

[ref122] Reich N. G. Shrestha S. King A. A. Rohani P. Lessler J. Kalayanarooj S.. (2013). Interactions between serotypes of dengue highlight epidemiological impact of cross-immunity. J. R. Soc. Interface 10:20130414. doi: 10.1098/rsif.2013.0414, PMID: 23825116 PMC3730691

[ref123] Rivera L. Biswal S. Saez-Llorens X. Reynales H. Lopez-Medina E. Borja-Tabora C.. (2022). Three-year efficacy and safety of Takeda's dengue vaccine candidate (TAK-003). Clin. Infect. Dis. 75, 107–117. doi: 10.1093/cid/ciab864, PMID: 34606595 PMC9402653

[ref124] Rothman A. L. (2011). Immunity to dengue virus: a tale of original antigenic sin and tropical cytokine storms. Nat. Rev. Immunol. 11, 532–543. doi: 10.1038/nri3014, PMID: 21760609

[ref125] Russell P. K. Buescher E. L. Mccown J. M. Ordonez J. (1966). Recovery of dengue viruses from patients during epidemics in Puerto Rico and East Pakistan. Am. J. Trop. Med. Hyg. 15, 573–579. doi: 10.4269/ajtmh.1966.15.573 4957424

[ref126] Sabchareon A. Wallace D. Sirivichayakul C. Limkittikul K. Chanthavanich P. Suvannadabba S.. (2012). Protective efficacy of the recombinant, live-attenuated, CYD tetravalent dengue vaccine in Thai schoolchildren: a randomised, controlled phase 2b trial. Lancet 380, 1559–1567. doi: 10.1016/S0140-6736(12)61428-7, PMID: 22975340

[ref127] Saied K. G. Al-Taiar A. Altaire A. Alqadsi A. Alariqi E. F. Hassaan M. (2015). Knowledge, attitude and preventive practices regarding dengue fever in rural areas of Yemen. Int. Health 7, 420–425. doi: 10.1093/inthealth/ihv021, PMID: 25858280

[ref128] Salje H. Cummings D. A. T. Rodriguez-Barraquer I. Katzelnick L. C. Lessler J. Klungthong C.. (2018). Reconstruction of antibody dynamics and infection histories to evaluate dengue risk. Nature 557, 719–723. doi: 10.1038/s41586-018-0157-4, PMID: 29795354 PMC6064976

[ref129] Salje H. Paul K. K. Paul R. Rodriguez-Barraquer I. Rahman Z. Alam M. S.. (2019). Nationally-representative serostudy of dengue in Bangladesh allows generalizable disease burden estimates. eLife 8:e42869. doi: 10.7554/eLife.42869, PMID: 30958263 PMC6513551

[ref130] Sangkaew S. Ming D. Boonyasiri A. Honeyford K. Kalayanarooj S. Yacoub S.. (2021). Risk predictors of progression to severe disease during the febrile phase of dengue: a systematic review and meta-analysis. Lancet Infect. Dis. 21, 1014–1026. doi: 10.1016/S1473-3099(20)30601-0, PMID: 33640077 PMC8240557

[ref131] Sankaradoss A. Jagtap S. Nazir J. Moula S. E. Modak A. Fialho J.. (2022). Immune profile and responses of a novel dengue DNA vaccine encoding an EDIII-NS1 consensus design based on indo-African sequences. Mol. Ther. 30, 2058–2077. doi: 10.1016/j.ymthe.2022.01.013, PMID: 34999210 PMC8736276

[ref132] Sawant J. Patil A. Kurle S. (2023). A review: understanding molecular mechanisms of antibody-dependent enhancement in viral infections. Vaccines (Basel) 11:1240. doi: 10.3390/vaccines11071240 37515055 PMC10384352

[ref133] Seesen M. Jearanaiwitayakul T. Limthongkul J. Midoeng P. Sunintaboon P. Ubol S. (2023). A bivalent form of nanoparticle-based dengue vaccine stimulated responses that potently eliminate both DENV-2 particles and DENV-2-infected cells. Vaccine 41, 1638–1648. doi: 10.1016/j.vaccine.2023.01.062, PMID: 36740559

[ref134] Seng C. M. Setha T. Nealon J. Socheat D. Chantha N. Nathan M. B. (2008). Community-based use of the larvivorous fish *Poecilia reticulata* to control the dengue vector *Aedes aegypti* in domestic water storage containers in rural Cambodia. J. Vector Ecol. 33, 139–144. doi: 10.3376/1081-1710(2008)33[139:CUOTLF]2.0.CO;2, PMID: 18697316

[ref135] Sharif N. Sharif N. Khan A. Dey S. K. (2024). The epidemiologic and clinical characteristics of the 2023 dengue outbreak in Bangladesh. Open Forum Infect. Dis. 11:ofae066. doi: 10.1093/ofid/ofae066 38390460 PMC10883285

[ref136] Sharmin S. Glass K. Viennet E. Harley D. (2018). Geostatistical mapping of the seasonal spread of under-reported dengue cases in Bangladesh. PLoS Negl. Trop. Dis. 12:e0006947. doi: 10.1371/journal.pntd.0006947, PMID: 30439942 PMC6264868

[ref137] Sharmin S. Viennet E. Glass K. Harley D. (2015). The emergence of dengue in Bangladesh: epidemiology, challenges and future disease risk. Trans. R. Soc. Trop. Med. Hyg. 109, 619–627. doi: 10.1093/trstmh/trv067, PMID: 26333430

[ref138] Sheng Z. Chen H. Feng K. Gao N. Wang R. Wang P.. (2019). Electroporation-mediated immunization of a candidate DNA vaccine expressing dengue virus serotype 4 prM-E antigen confers long-term protection in mice. Virol. Sin. 34, 88–96. doi: 10.1007/s12250-019-00090-8, PMID: 30790202 PMC6420567

[ref139] Shepard D. S. Undurraga E. A. Halasa Y. A. Stanaway J. D. (2016). The global economic burden of dengue: a systematic analysis. Lancet Infect. Dis. 16, 935–941. doi: 10.1016/S1473-3099(16)00146-8, PMID: 27091092

[ref140] Shirin T. Muraduzzaman A. K. M. Alam A. N. Sultana S. Siddiqua M. Khan M. H.. (2019). Largest dengue outbreak of the decade with high fatality may be due to reemergence of DEN-3 serotype in Dhaka, Bangladesh, necessitating immediate public health attention. New Microbes New Infect. 29:100511. doi: 10.1016/j.nmni.2019.01.007, PMID: 30937172 PMC6426716

[ref141] Sim S. Ng L. C. Lindsay S. W. Wilson A. L. (2020). A greener vision for vector control: the example of the Singapore dengue control programme. PLoS Negl. Trop. Dis. 14:e0008428. doi: 10.1371/journal.pntd.0008428, PMID: 32853197 PMC7451545

[ref142] Simmons C. P. Chau T. N. Thuy T. T. Tuan N. M. Hoang D. M. Thien N. T.. (2007). Maternal antibody and viral factors in the pathogenesis of dengue virus in infants. J. Infect. Dis. 196, 416–424. doi: 10.1086/519170, PMID: 17597456 PMC4333207

[ref143] Simmons C. P. Farrar J. J. Nguyen V. Wills B. (2012). Dengue. N. Engl. J. Med. 366, 1423–1432. doi: 10.1056/NEJMra1110265 22494122

[ref144] Srikiatkhachorn A. (2009). Plasma leakage in dengue haemorrhagic fever. Thromb. Haemost. 102, 1042–1049. doi: 10.1160/TH09-03-0208, PMID: 19967133 PMC5527705

[ref145] Suzuki K. Phadungsombat J. Nakayama E. E. Saito A. Egawa A. Sato T.. (2019). Genotype replacement of dengue virus type 3 and clade replacement of dengue virus type 2 genotype cosmopolitan in Dhaka, Bangladesh in 2017. Infect. Genet. Evol. 75:103977. doi: 10.1016/j.meegid.2019.103977 31351235

[ref146] Tayal A. Kabra S. K. Lodha R. (2023). Management of dengue: An updated review. Indian J. Pediatr. 90, 168–177. doi: 10.1007/s12098-022-04394-8, PMID: 36574088 PMC9793358

[ref147] Teo A. Chua C. L. L. Chia P. Y. Yeo T. W. (2021). Insights into potential causes of vascular hyperpermeability in dengue. PLoS Pathog. 17:e1010065. doi: 10.1371/journal.ppat.1010065, PMID: 34882753 PMC8659665

[ref148] Teo A. Tan H. D. Loy T. Chia P. Y. Chua C. L. L. (2023a). Correction: understanding antibody-dependent enhancement in dengue: are afucosylated IgG1s a concern? PLoS Pathog. 19:e1011736. doi: 10.1371/journal.ppat.1011736, PMID: 37851611 PMC10584112

[ref149] Teo A. Tan H. D. Loy T. Chia P. Y. Chua C. L. L. (2023b). Understanding antibody-dependent enhancement in dengue: are afucosylated IgG1s a concern? PLoS Pathog. 19:e1011223. doi: 10.1371/journal.ppat.1011223, PMID: 36996026 PMC10062565

[ref150] Thomas S. J. (2023). Is new dengue vaccine efficacy data a relief or cause for concern? NPJ Vaccines 8:55. doi: 10.1038/s41541-023-00658-2 37061527 PMC10105158

[ref151] Thomas L. Verlaeten O. Cabie A. Kaidomar S. Moravie V. Martial J.. (2008). Influence of the dengue serotype, previous dengue infection, and plasma viral load on clinical presentation and outcome during a dengue-2 and dengue-4 co-epidemic. Am. J. Trop. Med. Hyg. 78, 990–998. doi: 10.4269/ajtmh.2008.78.990, PMID: 18541782

[ref152] Tricou V. Essink B. Ervin J. E. Turner M. Escudero I. Rauscher M.. (2023). Immunogenicity and safety of concomitant and sequential administration of yellow fever YF-17D vaccine and tetravalent dengue vaccine candidate TAK-003: A phase 3 randomized, controlled study. PLoS Negl. Trop. Dis. 17:e0011124. doi: 10.1371/journal.pntd.0011124, PMID: 36888687 PMC9994689

[ref153] Tricou V. Yu D. Reynales H. Biswal S. Saez-Llorens X. Sirivichayakul C.. (2024). Long-term efficacy and safety of a tetravalent dengue vaccine (TAK-003): 4.5-year results from a phase 3, randomised, double-blind, placebo-controlled trial. Lancet Glob. Health 12, e257–e270. doi: 10.1016/S2214-109X(23)00522-3 38245116

[ref154] Troost B. Smit J. M. (2020). Recent advances in antiviral drug development towards dengue virus. Curr. Opin. Virol. 43, 9–21. doi: 10.1016/j.coviro.2020.07.009, PMID: 32795907

[ref155] Utarini A. Indriani C. Ahmad R. A. Tantowijoyo W. Arguni E. Ansari M. R.. (2021). Efficacy of Wolbachia-infected mosquito deployments for the control of dengue. N. Engl. J. Med. 384, 2177–2186. doi: 10.1056/NEJMoa2030243 34107180 PMC8103655

[ref156] Van Den Berg H. Zaim M. Yadav R. S. Soares A. Ameneshewa B. Mnzava A.. (2012). Global trends in the use of insecticides to control vector-borne diseases. Environ. Health Perspect. 120, 577–582. doi: 10.1289/ehp.1104340, PMID: 22251458 PMC3339467

[ref157] Vasilakis N. Cardosa J. Diallo M. Sall A. A. Holmes E. C. Hanley K. A.. (2010). Sylvatic dengue viruses share the pathogenic potential of urban/endemic dengue viruses. J. Virol. 84, 3726–3728; author reply 3727-3728. doi: 10.1128/JVI.02640-09, PMID: 20212326 PMC2838117

[ref158] Vaughn D. W. Green S. Kalayanarooj S. Innis B. L. Nimmannitya S. Suntayakorn S.. (2000). Dengue viremia titer, antibody response pattern, and virus serotype correlate with disease severity. J. Infect. Dis. 181, 2–9. doi: 10.1086/315215, PMID: 10608744

[ref159] Vicente C. R. Herbinger K. H. Froschl G. Malta Romano C. Cabidelle D. S. A. Cerutti Junior C. (2016). Serotype influences on dengue severity: a cross-sectional study on 485 confirmed dengue cases in Vitoria, Brazil. BMC Infect. Dis. 16:320. doi: 10.1186/s12879-016-1668-y, PMID: 27393011 PMC4938938

[ref160] Walsh M. R. Alam M. S. Pierce K. K. Carmolli M. Alam M. Dickson D. M.. (2023). Safety and durable immunogenicity of the TV005 tetravalent dengue vaccine, across serotypes and age groups, in dengue-endemic Bangladesh: a randomised, controlled trial. Lancet Infect. Dis. 24, 150–160. doi: 10.1016/S1473-3099(23)00520-0 37776876 PMC11267251

[ref161] Wang W. K. Chen H. L. Yang C. F. Hsieh S. C. Juan C. C. Chang S. M.. (2006). Slower rates of clearance of viral load and virus-containing immune complexes in patients with dengue hemorrhagic fever. Clin. Infect. Dis. 43, 1023–1030. doi: 10.1086/507635, PMID: 16983615

[ref162] Wang T. T. Sewatanon J. Memoli M. J. Wrammert J. Bournazos S. Bhaumik S. K.. (2017). IgG antibodies to dengue enhanced for FcgammaRIIIA binding determine disease severity. Science 355, 395–398. doi: 10.1126/science.aai8128, PMID: 28126818 PMC5557095

[ref163] Weeratunga P. Rodrigo C. Fernando S. D. Rajapakse S. (2017). Control methods for Aedes albopictus and *Aedes aegypti*. Cochrane Database Syst. Rev. 2017:CD012759. doi: 10.1002/14651858.CD012759

[ref164] Wegman A. D. Fang H. Rothman A. L. Thomas S. J. Endy T. P. Mccracken M. K.. (2021). Monomeric IgA antagonizes IgG-mediated enhancement of DENV infection. Front. Immunol. 12:777672. doi: 10.3389/fimmu.2021.777672, PMID: 34899736 PMC8654368

[ref165] Wegman A. D. Waldran M. J. Bahr L. E. Lu J. Q. Baxter K. E. Thomas S. J.. (2023). DENV-specific IgA contributes protective and non-pathologic function during antibody-dependent enhancement of DENV infection. PLoS Pathog. 19:e1011616. doi: 10.1371/journal.ppat.1011616, PMID: 37639455 PMC10491401

[ref166] Whitehead S. S. (2016). Development of TV003/TV005, a single dose, highly immunogenic live attenuated dengue vaccine; what makes this vaccine different from the Sanofi-Pasteur CYD vaccine? Expert Rev. Vaccines 15, 509–517. doi: 10.1586/14760584.2016.1115727, PMID: 26559731 PMC4956407

[ref167] Whitehead S. S. Durbin A. P. Pierce K. K. Elwood D. Mcelvany B. D. Fraser E. A.. (2017). In a randomized trial, the live attenuated tetravalent dengue vaccine TV003 is well-tolerated and highly immunogenic in subjects with flavivirus exposure prior to vaccination. PLoS Negl. Trop. Dis. 11:e0005584. doi: 10.1371/journal.pntd.0005584, PMID: 28481883 PMC5436874

[ref168] Wilder-Smith A. (2023). The dengue-in-Dhaka initiative: results from a phase 2 trial evaluating the TV005 tetravalent dengue vaccine in Bangladesh. Lancet Infect. Dis. 24, 112–113. doi: 10.1016/S1473-3099(23)00565-0 37776878

[ref169] Wilder-Smith A. (2024). TAK-003 dengue vaccine as a new tool to mitigate dengue in countries with a high disease burden. Lancet Glob. Health 12, e179–e180. doi: 10.1016/S2214-109X(23)00590-9, PMID: 38245106

[ref170] Wilder-Smith A. Hombach J. Ferguson N. Selgelid M. O'Brien K. Vannice K.. (2019). Deliberations of the strategic advisory Group of Experts on immunization on the use of CYD-TDV dengue vaccine. Lancet Infect. Dis. 19, e31–e38. doi: 10.1016/S1473-3099(18)30494-8 30195995

[ref171] Wilson A. L. Courtenay O. Kelly-Hope L. A. Scott T. W. Takken W. Torr S. J.. (2020). The importance of vector control for the control and elimination of vector-borne diseases. PLoS Negl. Trop. Dis. 14:e0007831. doi: 10.1371/journal.pntd.0007831, PMID: 31945061 PMC6964823

[ref172] Wollner C. J. Richner M. Hassert M. A. Pinto A. K. Brien J. D. Richner J. M. (2021). A dengue virus serotype 1 mRNA-LNP vaccine elicits protective immune responses. J. Virol. 95:e02482-20. doi: 10.1128/JVI.02482-20, PMID: 33762420 PMC8315947

[ref173] World Health Organization (2023a). Dengue-Bangladesh. Updated on 11 August, 2023. Available at: https://www.who.int/emergencies/disease-outbreak-news/item/2023-DON481#:~:text=However%2C%20DENV2%20has%20been%20identified,infection%20with%20a%20heterologous%20serotype (Accessed October 20, 2023).

[ref174] World Health Organization (2023b). Dengue-Global situation. Updated on 21 December, 2023. Available at: https://www.who.int/emergencies/disease-outbreak-news/item/2023-DON498#:~:text=In%20particular%2C%20India%2C%20Indonesia%2C,larger%20number%20of%20dengue%20cases%20 (Accessed March 8, 2024).

[ref175] Yang X. Quam M. B. M. Zhang T. Sang S. (2021). Global burden for dengue and the evolving pattern in the past 30 years. J. Travel Med. 28:taab146. doi: 10.1093/jtm/taab146, PMID: 34510205

[ref176] Yew Y. W. Ye T. Ang L. W. Ng L. C. Yap G. James L.. (2009). Seroepidemiology of dengue virus infection among adults in Singapore. Ann. Acad. Med. Singap. 38, 667–675. doi: 10.47102/annals-acadmedsg.V38N8p667, PMID: 19736569

[ref177] Young E. Yount B. Pantoja P. Henein S. Meganck R. M. Mcbride J.. (2023). A live dengue virus vaccine carrying a chimeric envelope glycoprotein elicits dual DENV2-DENV4 serotype-specific immunity. Nat. Commun. 14:1371. doi: 10.1038/s41467-023-36702-x, PMID: 36914616 PMC10009830

[ref178] Yunus E. M. Bangali A. M. Mahmood M. A. H. Rahman M. M. Chowdhury A. R. Talukder K. R. (2001). Dengue outbreak 2000 in Bangladesh: from speculation to reality and exercises. Dengue Bull. 25, 15–20.

[ref179] Zhang M. Sun J. Li M. Jin X. (2020). Modified mRNA-LNP vaccines confer protection against experimental DENV-2 infection in mice. Mol. Ther. Methods Clin. Dev. 18, 702–712. doi: 10.1016/j.omtm.2020.07.013, PMID: 32913878 PMC7452130

